# Pop‐Inference: An educational application to evaluate statistical differences among populations

**DOI:** 10.1002/ece3.4010

**Published:** 2018-05-04

**Authors:** Julio Arrontes

**Affiliations:** ^1^ Departamento de Biología de Organismos y Sistemas University of Oviedo Oviedo Spain

**Keywords:** demography, life table response experiments, power, projection matrix, statistical inference

## Abstract

Pop‐Inference is an educational tool designed to help teaching of hypothesis testing using populations. The application allows for the statistical comparison of demographic parameters among populations. Input demographic data are projection matrices or raw demographic data. Randomization tests are used to compare populations. The tests evaluate the hypothesis that demographic parameters differ among groups of individuals more that should be expected from random allocation of individuals to populations. Confidence intervals for demographic parameters are obtained using the bootstrap. Tests may be global or pairwise. In addition to tests on differences, one‐way life table response experiments (LTRE) are available for random and fixed factors. Planned (a priori) comparisons are possible. Power of comparison tests is evaluated by constructing the distribution of the test statistic when the null hypothesis is true and when it is false. The relationship between power and sample size is explored by evaluating differences among populations at increasing population sizes, while keeping vital rates constant.

## 1 INTRODUCTION

Pop‐Inference is an application written in MATLAB (The MathWorks Inc., Natick, MA, USA) code that uses randomization tests and the bootstrap to compare population parameters extracted from two or more populations. The application was written, and has been used, as an educational tool in postgraduate courses of population biology at the University of Oviedo, Spain. Students are often puzzled by the possibility of comparing population parameters, such as the population growth rate, using one single observation of the parameter per population. They feel that comparing just single numbers is wrong and that they need some estimation of the variability of the demographic parameters. Even among PhD students, it is not rare the feeling that several replicate estimates of the parameter should be independently obtained to compare populations. However, the comparison of population using single observations of the growth rates is common (e.g., Angert, 2006; Bruna & Oli, 2005). It is not conceptually different to compare, for example, two sample means. A sample mean is obtained after applying a simple formula to magnitudes obtained from individuals in a sample. A population growth rate is a nonlinear function of the fates of a sample of individuals during the projection interval. Of course, they are different because one is an individual property and the other is a population property, but both parameters are magnitudes averaged across the individuals obtained after sampling a population. And most important, confidence intervals may be constructed, using the information in the sample.

A significant difference in a demographic parameter implies that populations differ in their patterns of survival, growth, and/or reproduction. The reverse is not necessarily true (for a discussion on the logics and interpretation of experiments, see Underwood, 1990). Two populations may exhibit identical values for some demographic parameter with contrasting vital rates (e.g., opposite values for survival and fecundity). Two populations differ if they have different survival, growth, and/or reproduction patterns, that is, a different composition of individual life histories. An individual life history is defined by the stage at the start and at the end of the projection interval (death is considered a stage) of the individual and the number of recruits produced. After nonsignificant tests, if two populations do not have different composition of life histories, we cannot reject the hypothesis of a common origin of individuals. However, we cannot say they come from the same population or from two populations subject to the same environmental constraints.

Pop‐Inference tests the hypothesis that the observed differences between populations are beyond the expected differences from sets of individuals sampled at random from a single population. The application obtains the distribution of differences among populations under the null hypothesis of a common origin for individuals and then compares the observed difference with this distribution. The program also estimates the power of the comparison tests and obtains the confidence intervals for a set of demographic parameters. The application relies in randomization tests (Edgington, 1980; Manly, 2007) and the bootstrap (Efron & Tibshirani, 1993). Details of their application to matrix population models may be found in Caswell (2001). See a detailed description in Appendix S1.

The interest of the application is not limited to the numerical output of inferential tests, the *p*‐values. The major objective is the understanding of the whole process of decision taking involving populations. As important as the *p*‐values are (i) the analysis of the distributions of the test statistics under true and false null hypotheses and (ii) the analysis of the effects of increased densities on power, *p*‐values, and confidence intervals. Pop‐Inference may be used by students in a dual way. At a first stage, to learn how to analyze and interpret differences among populations and then to analyze differences among populations using data from their own projects.

Tests on differences in demographic parameters, construction of confidence intervals, and life table response experiments (LTRE) are routinely performed using both randomization tests and the bootstrap (e.g., Angert, 2006; Bruna & Oli, 2005; Cerná & Münzbergová, 2013; Münzbergová, 2007). However, in most cases, authors use ad hoc, home‐made, scripts not easily available, and at any case, difficult to use with students. Packages with functions and routines for demographic analysis, covering part of the topics exposed here, are indeed available. For example, the popbio package (Stubben & Milligan, 2007), but again, they are not friendly for their classroom use. To the best of my knowledge, no application or script is available to estimate power of tests comparing demographic parameters.

Inspiration came from Caswell (2001) and Manly (2007). Caswell (2001) provided pieces of MATLAB code to estimate population parameters, the algorithms for randomization tests and the bootstrap, the calculation of confidence intervals, the equations and discussion for the LTRE, and the steps to construct projection matrices from pre‐ and postbreeding censuses.

## 2 NATURE OF DATA

Input data may be a standard projection matrix or raw demographic data. If data come as projection matrices, fecundity matrices and transitions matrices must be specified for each population (see definitions in Caswell, 2001). Additional data include the number of individuals studied, and the nature of the census (pre‐ or postbreeding). The number of individuals used to construct the matrix is a critical variable. It is not possible to compare two or more populations using their projection matrices if the numbers of individuals used for their construction are not available. For any group of nonidentical matrices, significant differences always appear if a sufficiently large number of individuals is assumed (or was used to obtain the matrices). This is not different to ordinary sampling design and hypothesis testing (Snedecor & Cochran, 1980).

Raw demographic data describe the fate of every individual in the population during the projection interval in terms of survival, growth, and reproduction. For each stage, raw demographic data must always include the number of individuals dying during the projection interval and the number of individuals remaining, promoting to higher classes or going back to previous stages. Depending on the available information on reproduction, different classes of raw data are possible. Parents for every new individual in the population may be identified and their destinations or origins are known, or parents cannot be identified. For the latter case, three types are allowed in the application: type 1, reproductives are not identified (anonymous reproduction in Caswell, 2001); type 2, reproductives are identified but their origin or destination is not known and; type 3, reproductives are identified and their origin or destination is known. The distinction is relevant, as it is critical to obtain average fecundity and to allocate individuals to different histories for bootstrapping (Appendix S2).

Data may come from two different sampling designs: pure random sampling of individuals or sampling a fixed number of individuals in each stage. In the first case, the proportion of each stage in the sample reflects their proportion in the population and is an estimation of the population stage structure. If the number of individuals sampled in at least one stage is fixed, collected data do not reflect the population stage structure. This is relevant for the bootstrap and randomization tests. For pure random sampling, individuals are resampled with no restrictions. For fixed numbers per stage, resampling is restricted to each stage. More uncertainty and larger confidence intervals are obtained in the first case.

The timing for the collection of demographic data has an influence on the estimation of fecundities in the projection matrices. Fecundity is the average number of new individuals produced by an individual during the projection interval. Therefore, it is a combination of the maternity function (the average number of recruits produced by a reproductive female/individual) and survival. As a simplification, it is often assumed that data are collected immediately before (prebreeding) or after the reproductive season (postbreeding censuses). See Akçakaya, Burgman, and Ginzburg (1999) for the implications in matrix construction. No mortality during the reproductive period is considered. This is not realistic, but in most cases data can be accommodated to any of these approaches. Mortality of adults and recruits during the breeding season appears in the data as a reduction of fecundity. In prebreeding censuses, newly born individuals must survive the entire projection interval to be counted. In postbreeding censuses, individuals must survive during the whole projection interval, to the onset of the next reproductive period, and then, to reproduce with the fertility associated with their new stage (see also Caswell, 2001).

## 3 DESCRIPTIVE INFORMATION ON POPULATIONS

Descriptive information for each population includes six demographic parameters that will be used to compare populations and sensitivity and elasticity matrices. Except for the composition of histories, for each parameter, the application calculates the observed value, the 90% and 95% confidence intervals, and the simulated median. Confidence intervals are calculated using bootstrap techniques (Efron & Tibshirani, 1993). See Appendix S3 for a complete output.

- The asymptotic population growth rate, λ. It is the rate of population increase when the population is at the stable stage structure. The rate is referred to a fixed time interval, the projection interval. The growth rate is calculated as the dominant eigenvalue of the population matrix (e.g., Vandermeer & Goldberg, 2003).
- The net reproductive rate (*R*_0_), defined as the average number of new individuals produced by a newly born individual during its lifetime. *R*_0_ is calculated as the dominant eigenvalue of the matrix **R**, which is obtained as **R = FN**, where **N** is the fundamental matrix whose elements are the expected times spent at each stage by individuals in the population, and **F** is a fecundity matrix with the expected number of recruits of each class produced by time step (Caswell, 2001).
- The generation time (*T*) is the time needed by the population to increase by a factor of *R*_0_. Because of λ^T^ = *R*_0_, the generation time is estimated as *T* = ln *R*_0_/ln λ (Caswell, 2001).
- The stable stage structure (SSS) gives the proportion of individuals in each stage that any initial distribution of individuals converges to (Akçakaya et al., 1999). On reaching SSS, the population will keep a constant proportion of individuals at each stage or age through time. At SSS, the observed rate of growth also remains constant and equals λ. The SSS is calculated as the right eigenvector of the population matrix corresponding to the dominant eigenvalue (Caswell, 2001). The SSS is expressed as the proportion of each stage in the population. If sampling was at random, the observed stage distribution is also given. The distance between the observed and the stable stage distributions is calculated using the Keyfitz's distance (Keyfitz & Caswell, 2005). The statistical significance of the distance to SSS is obtained by a randomization test. If the number of individuals studied in each stage is in some way controlled by the experimenter by setting minimum, maximum, or fixed numbers of individuals, the distance to SSS and its significance are meaningless quantities and are not calculated.
- The reproductive value (RV) is the Fisherian concept of age‐specific reproductive value generalized by Goodman (1968). Intuitively, the RV represents the contribution of each stage to all other stages. The RV is calculated as the left eigenvector of the population matrix corresponding to the dominant eigenvalue (e.g., Lanciani, 1998; Vandermeer & Goldberg, 2003). The RV is expressed as the proportional contribution of each stage to the abundance of all stages (Caswell, 2001).
- The collection of individual histories. An individual history is a summary of the events of the life cycle associated with the individuals in the population. The individual history describes the pattern of survival, growth, and reproduction affecting each individual. The history specifies whether the individual survives or not and the destination class if it does and gives the number of recruits produced during the projection interval and the stages to which new individual recruit. To implement the bootstrap and randomization tests, Caswell (2001) suggested the construction of auxiliary matrices whose columns represent the individuals with the associated events during the projection interval. This is explained more in detail in Appendix S1.
- Sensitivity and elasticity matrices describe the changes in the asymptotic population growth rate, λ, as a consequence of changes in matrix entries (Caswell, 2001). Sensitivity describes absolute changes in λ due to of absolute changes in vital rates. Elasticity describes proportional changes in λ in response to proportional changes in vital rates. Practical limitations in the interpretation of sensitivity and elasticity matrices are discussed by Akçakaya et al. (1999). They are not used to compare populations.

Pop‐Inference may also perform life table response experiments (LTRE). LTRE evaluate how variability in vital rates contributes to the observed variability in λ. LTRE for both random and fixed effects are available, but only for one‐way designs. An example of the use of LTRE is in Zuidema, De Kroon, and Werger (2007). Details for computation were extracted from Caswell (2001). Sometimes, sensitivity or elasticity analysis is performed simultaneously to LTRE for a better evaluation of management strategies (e.g., Zuidema et al., 2007).

## 4 COMPARING POPULATIONS

Nonoverlapping confidence intervals have been used to evaluate differences in demographic parameters (Bruna & Oli, 2005). However, to test hypotheses about demographic parameters, randomization tests are a better option and are the most commonly used method (e.g., Angert, 2006; Brault & Caswell, 1993). Randomization tests are described in detail in the books by Edgington (1980), Manly (2007), and Caswell (2001). Tests in Pop‐Inference include global and pairwise comparisons and evaluate the null hypothesis that all populations in the study have a common origin. In other words, that the observed differences among populations should be due to sampling error associated with the random selection of individuals (with their associated histories). A significant global test indicates that at least one of the populations differ from the others, as the observed differences are beyond the expected random allocation of individuals to populations, but it does not identify the population(s) differing.

Test statistics differ in global or pairwise tests and depending on the nature of the demographic parameter (scalar or vectorial). The λ, *R*_0_ and *T* are scalar parameters. SSS, RV and the collection of individual histories are vectorial parameters. The three vectors are probability vectors (summing up to 1). The four test statistics below are different ways to express differences among a set of values. A survey of distance indices with comments on their relevance in different scenarios is given in Cha (2007).

For scalar population parameters in pairwise comparisons, the test statistic is the absolute difference between the two magnitudes,

$$d_{w}=\left|x_{1}-x_{2}\right|$$

where *x*_1_ and *x*_2_ are the parameter values for populations 1 and 2, respectively. For global comparisons, the test statistic is the sum of squares of the parameter,

$$d_{g}=\sum_{i=1}^{n}{\left(x_{i}-\overset{¯}{x}\right)}^{2}$$

where *n* is the number of populations, *x*_*i*_ is the parameter value for population *i* and $\overset{¯}{x}$ is the average parameter across populations.

For vectorial parameters in pairwise comparisons, the test statistic is

$$D_{w}=\frac{1}{2}\sum_{j=1}^{m}\left|p_{1j}-p_{2j}\right|$$

where *m* is the number of stages if SSS or RV are being compared among populations or the number of distinct individual histories, *p*_1*j*_ and $p_{2j}$ are the proportions for stage or history *j* in populations 1 or 2*. D*_*w*_ is a standard measure of the difference of two probability vectors and also is the Keyfitz's Delta (Keyfitz & Caswell, 2005). *D*_*w*_ takes values between 0, when both vectors are identical, and 1, when no overlap exists among the two vectors.

For global comparisons, the test statistic is

$$D_{g}=\frac{1}{2(n-1)}\sum_{j=1}^{m}\sum_{i=1}^{n}\left|p_{\mathrm{ij}}-{\overset{¯}{p}}_{.j}\right|$$

where *m* and *n* are as above, *p*_*ij*_ is the proportion of stage or history *j* in population *i* and ${\overset{¯}{p}}_{.j}$ is the mean proportion of stage or history *j* across populations. *D*_*g*_ is an estimation of average distance between individual populations and the mean parameter calculated across populations. *D*_*w*_ and *D*_*g*_ are arithmetically equivalent when the number of populations is 2. *D*_*g*_ takes values between 0 and 1.

**Figure 1:**
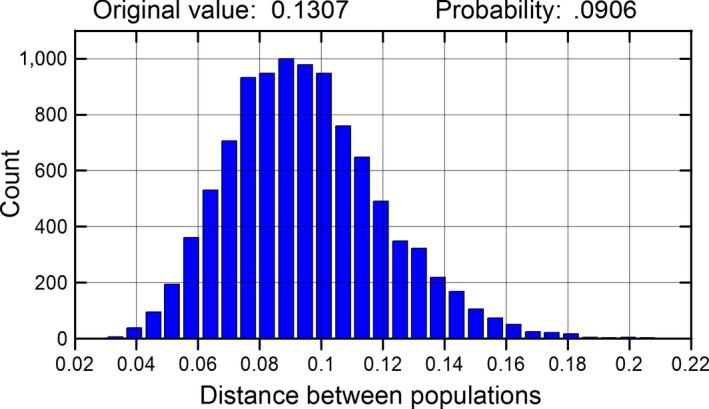
Distribution of the differences in the composition of individual histories after 10,000 permutations of individuals of two populations (populations 2 and 3 in Appendix S3). Permutations simulate random allocation of individuals to two samples taken from the same population and give the distribution of the test statistic under the null hypothesis of a common origin for both populations. The proportion of permutations with a test statistic larger than the original was 0.0906, and therefore, the original difference (0.1307) was not statistically significant at α = 0.05

The graphical output for comparison of populations includes the distribution of the test statistics under a true null hypothesis (Fig. 1). This distribution can be safely ignored if the interest of the user is strictly limited to the *p*‐value associated with the test. For students, however, the distribution illustrates the rationale of inferential tests. The distribution of the test statistic is equivalent to the distribution of differences among populations that should be expected by chance, when both populations have a common origin. Comparison of the observed test statistic and the graphed distributions allows the students for the interpretation of the output of the randomization test, as it says how extreme is the observed value in relation to the expected value under the null hypothesis.

A large number of pairwise comparisons have an associated increase in the overall probability of type I error, that is, rejecting a true null hypothesis (Quinn & Keough, 2002). As corrections usually come at the cost of decreased power, no action is taken by the application to this respect. The user must be aware of the problem and interpret multiple tests with caution. Appropriate corrections of *p*‐values are available (Quinn & Keough, 2002).

Sometimes the hypothesis of interest involves differences among groups of populations, such as the comparison of a control population against all other or the comparison of groups of populations sharing some common property. Planned comparisons among these a priori defined groups of populations are possible. See Underwood (1997) for a definition, and discussion, of a priori tests. To create groups of populations, all individuals from populations being grouped collapse into a single group. New demographic parameters are extracted from this new and larger population. Note that no averaging of parameters from individual populations is carried out. Any number of comparisons among groups is possible: Comparisons may be orthogonal when each single population or group of populations is used only once in the comparisons or nonorthogonal. See Underwood (1997) for a discussion on the interpretation of orthogonal and nonorthogonal contrasts.

## 5 EVALUATION OF POWER

Nonsignificant results may appear because, in fact, populations do not differ or because power was small. Power of a statistical test is the probability of rejecting a false null hypothesis (or the probability of detecting a real difference) (Quinn & Keough, 2002). Power is positively related to sample size, the magnitude effect, and the significance level. An evaluation of power is important but seldom possible before the experiment. After the study, an evaluation of power may help to understand the output and gives some indications for future studies (Underwood, 1997).

**Figure 2:**
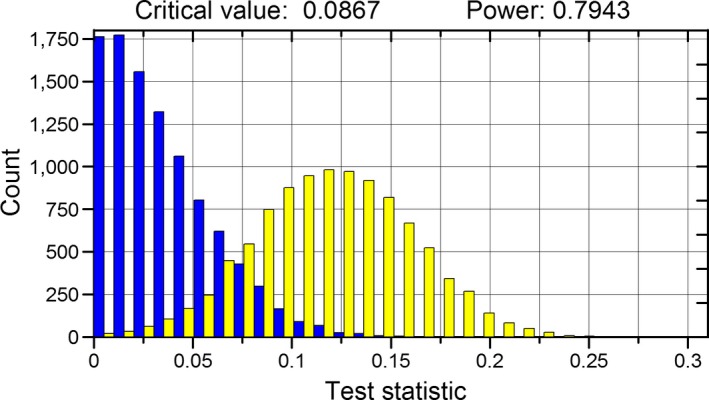
Distribution of the test statistic used to evaluate differences in λ between two populations when the null hypothesis is true (blue bars) and false (yellow bars). The critical value leaves out the 5% of larger values to the right of the distribution of the test statistic when the null hypothesis is true. Power, 0.7943, is the proportion of observations of the test statistic larger than the critical value when the null hypothesis is false. Distributions were obtained after 10,000 random permutations of individuals of two populations (populations 2 and 3 in Appendix S3)

Pop‐Inference calculates power of the tests used to compare populations using randomization and the bootstrap. First, the application obtains the distribution of the test statistic under the null hypothesis, assuming that all individuals have a common origin. Individuals from the populations being compared are pooled and then randomly allocated (without replacement) to the populations and the test statistic calculated again. The procedure is repeated a large number of times (say 10,000). From the distribution of simulated values, a critical value of the test statistic (leaving out 5% of largest observations) is obtained. Now, the application obtains the distribution of the test statistic assuming that the null hypothesis is false. Populations are independently resampled with replacement (simulating a new sample from each original population) and the test statistics computed again from the bootstrap samples. The proportion of values from this distribution larger than the critical value obtained assuming a true null hypothesis is an estimation of power (Fig. 2). Graphs of the distribution of the test statistics under the true and false null hypothesis illustrate the whole process to evaluate power. The interpretation of these graphs can be found in almost every Statistics textbook (e.g., Sokal & Rohlf, 1981; Underwood, 1997).

**Figure 3:**
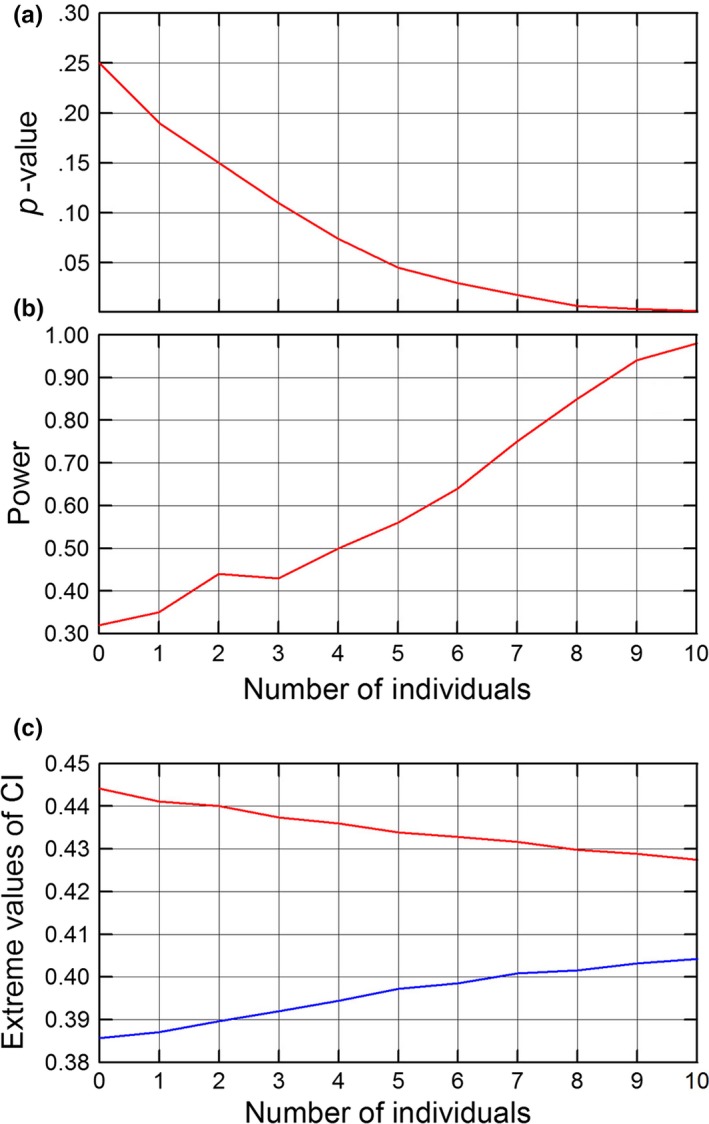
A simulation of expected results using samples of individuals of increasing size. Panels a and b are for the variation in the *p*‐value and statistical power of the comparison of the stable stage structure of populations 1 and 3 in Appendix S3. Lower panel (c) shows the expected variation in the 95% confidence interval (CI) for the proportion of individuals in one class of individuals at the stable stage structure (class 1 in population 3 of Appendix S3); blue line: lower limit of the CI; red line, upper limit. Numbers of individuals are coded. Number 0 is the original number of individuals. Each additional sample size is 20% larger than the previous one. In the three cases, 10,000 random permutations of individuals were used

Variation in power, in *p*‐values of tests and in the width of confidence intervals are explored at increasing densities (Fig. 3). They are of limited value when analyzing real population data, but are useful to students to understand the relationship between width of confidence intervals, power, and sample size (e.g., Quinn & Keough, 2002). In the three cases, the application evaluates *what would happen* if an increased number of individuals was sampled and identical vital rates obtained. This is not realistic because, by chance, different composition of vital rates should be obtained for each sample size. Simultaneously, increased sample sizes should render more precise composition of life histories. But the simulation would suggest approximate sample sizes to detect significant differences.

## 6 KNOWN LIMITATIONS AND UNCERTAINTIES

There are a few limitations imposed by the simplicity of the programming code: Only two tailed tests are available; data from all populations must be of the same type, with identical sampling designs, and populations must have the same number of stages.

Randomization tests evaluate differences between collections of individuals. They are not tests on populations (Manly, 2007, page 2). The tests tell if an observed difference between two collections of life histories might have appeared by chance. Extrapolation to populations requires that individuals (with their life histories) were collected independently and at random and are a fair representation of their respective populations.

Differences in the width of confidence intervals of vital rates among populations might influence the output of statistical tests. Obtaining extreme demographic parameters is more likely in populations with larger confidence intervals, and thus, the probability of erroneously detecting differences increases. In some way, this might be similar to the effect of heterogeneity of variances in ordinary hypothesis testing (Underwood, 1997). This aspect requires further attention.

As with any other inferential procedure, failing to reject the null hypothesis (i.e., nonsignificant tests) does not mean that populations are identical or are under identical environmental constraints (Underwood, 1990). Different processes might lead to similar or identical composition of life histories or identical demographic parameters.

## 7 EVALUATION OF Pop‐Inference AS A TEACHING TOOL

No formal evaluation tests were performed on the students’ response to the application. However, after 3 years of use, a few conclusions may be obtained. At the beginning of the course, students often have problems to extrapolate concepts of ordinary sampling design and hypothesis testing to populations. In general, replication, random sampling, and independence of observations are concepts already assimilated by postgraduate students. Their extrapolation to populations is facilitated by considering populations as collections of individual life histories. A particular life history may be seen as an individual “property,” as could be size or weight. Demographic parameters (including λ) are obtained after some working out of the life histories stored in auxiliary matrices. Conceptually, that is not very different to the extraction of well‐known parameters such as a variance.

Due to the lack of tabulated test statistics to compare demographic parameters, the need for randomization tests is well understood by the students. Randomization tests are made very intuitive by graphing the distribution of the test statistic under the true (and false, for power analyses) null hypothesis. Understanding the nature and interpretation of the randomization tests has a beneficial side effect. They reveal the importance of sample size and how individuals are sampled from populations. Students are given the keys to design better sampling programs to study demography of wild populations.

## CONFLICT OF INTEREST

None declared.

## AUTHOR CONTRIBUTION

This article has a single author who is responsible for the whole content.

## DATA ACCESSIBILITY

The application may be accessed from the Open Science Framework at https://osf.io/2ea6j/.
