# Supplementary material for: Pop‐Inference: An educational application to evaluate statistical differences among populations
Source: Ecol Evol. 2018 May 4;8(11):5224–30. doi: 10.1002/ece3.4010 (PMC6010711; doi:10.1002/ece3.4010)
Supplement: Supplementary file 1 [file ECE3-8-5224-s001.pdf]

GUIDE TO `Pop-Inference`

Julio Arrontes

Dept. Biología de Organismos y Sistemas. University of Oviedo. 33071-Oviedo, Spain

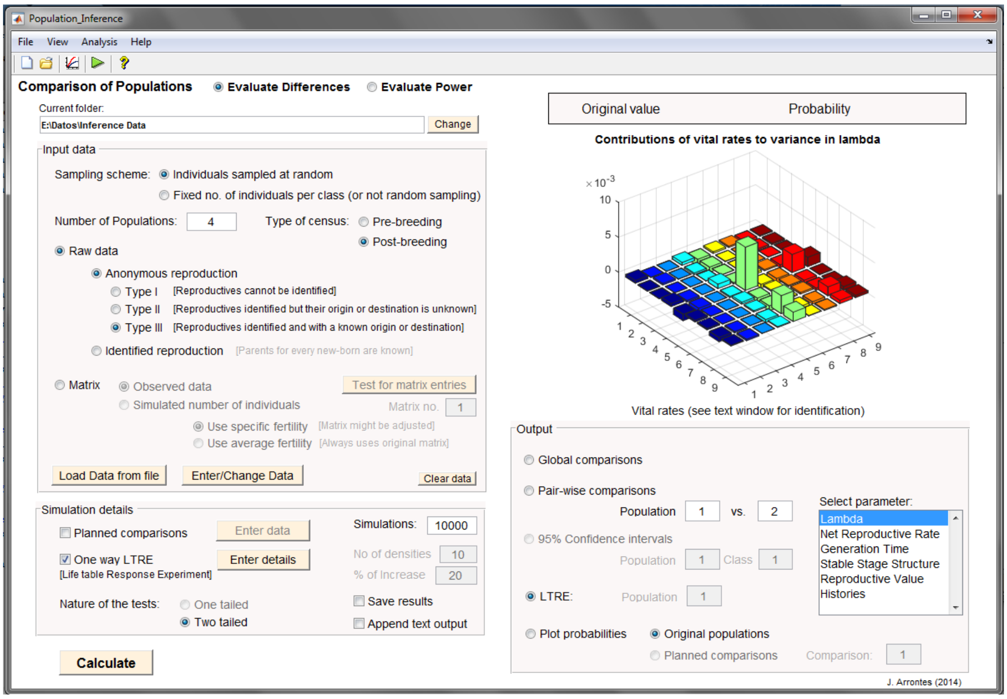

The application allows for two types of analyses: the evaluation of statistical differences among populations and an estimation of power of tests used to compare populations. Basic demographic information for each population and confidence intervals for relevant population parameters are also calculated.

Evaluation of differences: For any number of populations, the application extracts basic demographic information, obtains confidence intervals and estimates global and pairwise differences in six demographic variables:

(i) The asymptotic population growth rate (λ),
(ii) the net reproductive rate (R_0),
(iii) the generation time (T),
(iv) the stable stage structure (SSS),
(v) the reproductive value (RV) and,
(vi) the composition of individual histories during the projection interval.

Planned (*a priori*) comparisons are possible among any number of groups of pooled populations defined by the user or several pooled populations against a single population. Another option is to make a Life Table Response Experiment (LTRE), which is a sort of perturbation analysis (Caswell, 2001).

Evaluation of power: The application estimates power by constructing the distribution of the test statistics used to estimate the difference among populations when the null hypothesis is true and

when it is false. In addition, the application obtains confidence intervals and estimates differences among populations with increasing numbers of individuals in the populations. The projection matrix is kept constant and thus, changes in the width of confidence intervals or p-values may serve as an additional and indirect estimation of power.

The program uses the bootstrap to estimate confidence intervals. Global and pairwise differences among populations are evaluated using randomization methods. In both cases, the program constructs an auxiliary matrix containing the histories of all individuals in the populations during the projection interval. To calculate confidence intervals the auxiliary matrix is sampled with replacement many times and bootstrap estimates of the population parameters are obtained. For differences among populations, individual histories from the populations are grouped together in the same auxiliary matrix and, then, randomly allocated to populations. See Caswell (2001) and Manly (2007) for details on test statistics and for a full description of the procedures. A definition and explanation of the demographic parameters used may be found in Caswell (2001) and in any elementary Population Biology textbook (e.g. Hastings, 1997; Vandermeer & Goldberg, 2003; Rockwood, 2006). An individual history is defined by the class at the start of the interval, the class at the end (including death of the individual) and the number of recruits produced, with indication of the class to which new individuals recruit. In other words, histories summarize survival, growth and reproduction of individuals during the projection interval.

Inspiration for the development of this application came from the reading of Caswell's (2001) book. I benefited from concepts and the precise description of methods and algorithms. In addition, I included in the application code many pieces of Matlab® code described by Caswell (2001).

QUICK START GUIDE

1. Prepare files with demographic data. Files are text only and their format depends on the type of demographic information available. See sections **1.3** to **1.6.** You may also load data from the keyboard at a later stage.

2. Go to the application.

3. Select a working directory (if needed).

4. Specify the number of populations.

5. Specify sampling details: sampling scheme (**1.1**) and type of census (**1.2**). The latter is particularly relevant as the format of the files with data is different for pre- and post-breeding censuses. The sampling scheme is of secondary importance and only has a limited influence on the output.

6. Select the type of input data: raw demographic data (**1.3** and **1.4**) or projection matrix (**1.5**).

7. Select the appropriate options for each type of demographic data. For raw data, every option claims for a different data structure. For projection matrices selecting *Observed data* or *Simulated no. of individuals* is relevant for the data structure. At this stage, you may ignore tests for matrix entries and the fertility scheme used.

8. Load data from file or enter from keyboard (**1.8**).

9. Select the type of analysis: Evaluation of differences among populations or power analysis. You can switch the type of analysis any time.

10. Specify simulation details as needed (**2**). Common to both types of analyses are the number of simulations, the possibility to save results at runtime and how the text output is presented in the text window (**2.6**).

11. When comparing populations, if applicable, select the Planned comparisons (**2.3**) and/or LTRE options (**2.4**) and enter details for both analyses.

12. For power analysis, adjust the number of densities analysed and the increase in size (**2.6**).

13. Run the application.

14. See results as text output or as graphics (**3**).

INSTALLATION NOTES

Different options depending on the version of Matlab installed in the destination computer.

1) Use the application in Matlab

The application was written and tested in the R2015b version. It does not work in version R2012b. Not tested in other versions.

Copy the Matlab_Code folder, with all its files, into the current working directory. Run the application by calling Population_Inference in the command line.

Keep the Help folder in the same site as the *.p (or *.m) and *.fig files.

Code has been obfuscated to protect the functionality of the application against accidental changes. The original code can be obtained on request: arrontes@uniovi.es

2) Install the application as standalone

Use this option when Matlab is not installed or the version is not compatible. The application will run in Windows 64 bits only.

Run the Pop_Inference_Install.exe file. You will need administrator rights and an internet connection. By default, the application is installed at:

C:\Program Files\Uniovi\Pop_Inference

The installer will check if your computer has MATLAB Runtime, version 9.0 (R2015b), Windows 64-bit. If Matlab runtime is not in your computer, the installer will download it from the MathWorks Web site. Alter the installation of the Matlab runtime, the installer will resume the installation of the Pop_Inference. To run the application click on Pop_Inference.exe or create a desktop shortcut.

A working directory, *Inference data*, with two folders (*Data* and *Results*), will be created for the first time the application is accessed. By default, the working directory will be in the user data folder. A new working directory may be created anytime in another place by using the *Change* button. If the new destination folder has not the *Data* and/or *Results* folders, they are created by the application. You may give any name to the working directory, but keep the default sub-folders and names. If one or both folders it contains are deleted or renamed, the program may crash during import-export actions.

1. INPUT DATA

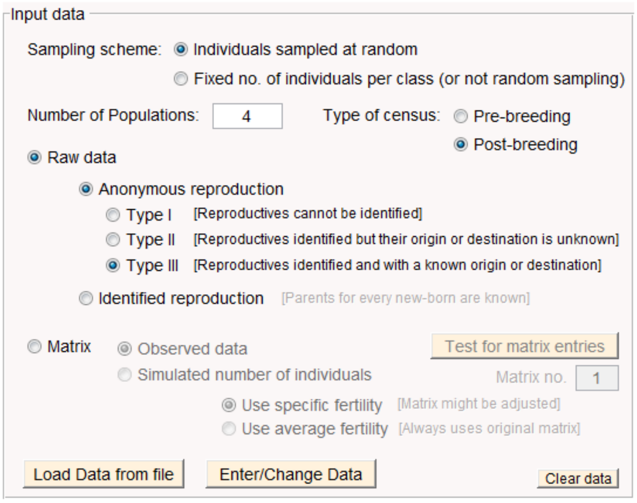

Data may come in many formats and cover almost any real situation. Data may be read by the program from a text only file (*txt* format). Data may also be entered form the keyboard.

Data may be given as an already constructed projection matrix or as raw demographic data. Selection of the nature of data is the first action after selecting the family of analyses. Then the number of populations being analyzed must be given in the *Populations* edit window.

1.1 Sampling scheme

This part refers to the sampling design used to study the original populations. It is relevant for the the bootstrap. Two options are considered:

1.1.1 Individuals sampled at random

This option should be selected when the individuals included in the study were sampled enterely at random. The number of individuals of each class studied was related to their relative abundance in the population. With this design the sample of individuals is considered to be a fair representation of the original population. When estimating confidence intervals using the bootstrap, individuals are resampled with replacement with no limitations. By chance, the number of individuals at each class may vary everytime the population is resampled.

1.1.2 Fixed no. of individuals per class

The number of individuals in at least one class was fixed beforehand and does not represent the relative abundance of the class in the population. Within each class, individuals are sampled at random. To estimate confidence intervals using the bootstrap under this design, individuals are resampled with replacement within each class. Now, the number of individuals at each class remains constant.

1.2 Type of census

Except when the *Identified reproductives* option is selected, the type of census must be indicated. If the wrong selection is done, sooner of later the program will crash.

Select *prebreeding censuses* when the population is studied immediately before reproduction. Individuals are counted, aged and sexed before the reproductive season. Recruits are counted when they are close to their first birthday.

Select *postbreeding censuses* if the population is studied after the reproductive season. Recruits are counted immediately after they arrive to the population.

Note: Structure of the data for a single population is explained below. See **section 1.6** for the organization of the *txt* files with all populations.

1.3 Raw data: Anonymous reproduction

There are two types of data: 1) Anonymous reproduction occurs when recruits cannot be assigned to any specific reproductive individual. 2) Identified reproductives, when the parents for every recruit are known.

The program distinguishes three types of anonymous reproduction:

1.3.1 Type I. Reproductive individuals cannot be identified

Every individual in the reproductive classes is considered as reproductive. An average fertility is estimated. For a population with *n* classes, raw data come in a matrix with *n* columns and *n+3* rows:

		Origin		
		Class1	Class 2	Class 3
Destination	Class 1	0	0	0
	Class 2	10	15	0
	Class 3	0	15	12
	Deaths	30	15	12
	Recruits	100	0	0
	Relative Fertility	0	1	2

C1 to *C3* are classes in the population. The number 10 at row 2 and column 1 means that 10 individuals in class 1 at the beginning of the projection interval were at class 2 at the end of the interval.

Deaths is the number of individuals at each class dying during the projection interval.

Recruits is the number of individuals entering the population during the projection interval. Here, 100 new individuals entering at class *C1*, but they might enter, for example, some at class *C1* and other at class *C2*.

Relative Fertility specifies differences in fertility among individuals at the reproductive classes. In the example, classes 2 and 3 are reproductive and individuals at class 3 are known to have a fertility twice of individuals at class 2. If no information on fertility is available, give 1 to any reproductive class. Relative fertility for non-reproductive classes is 0.

Formats are identical for pre- and post-breeding censuses. Data are in the *.txt* file without any label:

```
0    0    0
10   15   0
0    15   12
30   15   12
100  0    0
0    1    2
```

1.3.2 Type II. Reproductive individuals are identified but either the class of origin (in post-breeding censuses) or the destination (in pre-breeding censuses) are not known

A new row is added to the input table and file. The new row specifies the number of reproductive individuals at each class:

		Origin		
		Class 1	Class 2	Class 3
Destination	Class 1	0	0	0
	Class 2	10	15	0
	Class 3	0	15	12
	Deaths	30	15	12
	Recruits	100	0	0
	Relative Fertility	0	1	2
	Reproductive	0	9	16

Formats are identical for pre- and post-breeding censuses. The *.txt* file only contains numbers:

```
0    0    0
10   15   0
0    15   12
30   15   12
100  0    0
0    1    2
0    9    16
```

In the example, there were 9 reproductive individuals in class C2 and 16 in class C3, but their origin or destination classes are not known. In this situation, gross fertility is obtained by dividing the number of recruits by the number of reproductive animals. In case of a pre-breeding census, the destination of the 9 individuals of class 2 is proportionally allocated, in time t+1, to the C2, C3 and dead. The 16 of class C3 are allocated to C3 and dead (one third for each category). If it was a post-breeding census, the 9 individuals in class C2 could come from the 10 individuals promoting from class C1 to C2 or from the 15 staying in class C2. So, reproductives are allocated proportionally to these two origins. The same procedure is followed for the 16 reproductives in C3. One fraction comes from the 15 individuals promoting from C2 to C3 and the remaining from the 12 individuals staying in C3.

1.3.3 Type III. Reproductive individuals have either a known origin or a known destination

For **post-breeding** censuses in a population with *n* classes, the input data matrix has *2n+3* rows. The last *n* rows give the origin of the reproductives of each class:

		Origin		
		Class 1	Class 2	Class 3
Destination	Class 1	0	0	0
	Class 2	10	15	0
	Class 3	0	15	12
	Deaths	30	15	12
	Recruits	100	0	0
	Relative Fertility	0	1	2
	Reproductives C 1	0	0	0
	Reproductives C 2	4	5	0
	Reproductives C 3	0	9	7

The *.txt* file with the data:

```
0    0    0
10   15   0
0    15   12
30   15   12
100  0    0
0    1    2
0    0    0
4    5    0
0    9    7
```

Above, 4 of the reproductive individuals in class *C2* came from *C1* and 5 were in *C2*. Nine of the reproductive individuals in *C3* came from *C2* and 7 were in class *C3* one time step before.

If data come from a **pre-breeding** census, the table has *2*n+4* rows. The last *n+1* rows indicate the destination of the reproductive individuals during the projection interval, including death:

		Origin		
		Class 1	Class 2	Class 3
Destination	Class 1	0	0	0
	Class 2	10	15	0
	Class 3	0	15	12
	Deaths	30	15	12
	Recruits	100	0	0
	Relative Fertility	0	1	2
	End at C 1	0	0	0
	End at C 2	0	4	0
	End at C 3	0	3	9
	Dead	0	2	7

There are two rows for mortality. The 4th row is for the total number of deaths within each class of origin. The last row is only for reproductives.

The *.txt* file:

```
0    0    0
10   15   0
0    15   12
30   15   12
100  0    0
0    1    2
0    0    0
0    4    0
0    3    9
0    2    7
```

In the example, 9 individuals in C2 at time *t* reproduced, 4 of them survived the projection interval and remained in C2, 3 survived and jumped into C3 and 2 died. From 16 individuals reproducing in C3, 9 remained in the class and 7 died.

Important: Except for *Relative Fertility*, all data must be integers, as they are numbers of individuals.

A comment on fertility. Fertility and fecundity are two different demographic parameters which are sometimes confused. Here, fertility is considered as the ability of individuals to produce recruits. It is synonymous to maternity function and should be a consequence of the life cycle, anatomy and physiology of individuals. Fecundity is the realisation of fertility. It is the number of recruits left by an individual during some time interval.

1.4 Raw data: Identified reproductives

The parents of each new individual are known. Data are complete individual histories including origin class, the destination class and the number of new individuals produced (if reproductive). This is the preferred kind of data, as it gives the most accurate results. Data may come from pre- or post-breeding censuses. Because it is not relevant for the analyses, the options to select the type of census are disabled. Input data are a collection of histories, and are identical to the auxiliary matrix used for randomization tests and the bootstrap. For all other types of input data, the construction of the auxiliary matrix implies some averaging of vital rates. With identified

reproductives, individual histories are as observed in the field.

The number of rows in the input table is variable, depending on the number of classes to which the new individuals recruit. For example, 4 rows if all recruits appear in only one class (possibly the most common situation), 5 rows if new individuals recruit into 2 classes... The number of columns is the number of different individual histories defined during the projection interval (time *t*). The format is identical for both types of censuses (pre- and post-breeding).

The example below is for a population with 3 classes but with 2 different types of recruits. It could be for a post-breeding census. For example, history number 8 is for individuals which were in class 2 at start, died during the projection interval and reproduced, leaving 2 recruits in class 1. There were 15 individuals with this particular history. History 14, was for individuals starting in class 3, dying during the projection interval and not reproducing. There were 20 individuals with this history. If only one class of recruits existed, the 4th row would be missing and the table should have 4 rows.

Only integers should be in the table, as eantries are individuals or identify classes.

	Histories													
	1	2	3	4	5	6	7	8	9	10	11	12	13	14
Origin class	1	1	2	2	2	2	2	2	2	2	3	3	3	3
Destination class*	2	4	2	2	2	3	3	3	3	4	3	3	3	4
New individuals in class 1	0	0	0	1	1	0	1	2	2	0	0	1	2	0
New individuals in class 2	0	0	0	0	1	0	0	0	1	0	0	1	1	0
Number of individuals	15	30	9	12	20	10	0	15	3	20	10	12	19	20

*Class 4 is death (if a population has *n* classes, death is represented as class *n+1*)

No labels are used in the file:

```
1  1  2  2  2  2  2  2  2  2  3  3  3  3
2  4  2  2  2  3  3  3  3  4  3  3  3  4
0  0  0  1  1  0  1  2  2  0  0  1  2  0
0  0  0  0  1  0  0  0  1  0  0  1  1  0
15 30 9  12 20 10 0  15 3  20 10 12 19 20
```

Important: It may occur that some particular histories are present in some populations but not in others. Data for each population must contain all histories, present and absent, recorded in the study. Absent histories from a given population have an abundance of 0 individuals. This is the case for history 7 above. It was recorded in other population but not in this one: that is for an individual starting in class 2, ending in class 3 and leaving one recruit in class 1.

1.5 Matrix as input data

To evaluate differences among populations and obtain confidence intervals, fecundity and transition matrices must be obtained from the population matrix. If no information exists (particularly on the first element in the matrix), then assume that elements in the first row of the matrix are fecundities and all other elements transition probabilities. For the definition and shape of fecundity and transition matrices and the meaning of their elements, see any elementary Population Biology textbook.

There are two possibilities for matrices:

Observed data. The option should be selected when the number of individuals used to construct the projection matrix and their class structure is known.

Simulated number of individuals: It may occur that for one or more populations in the study, elements in the population matrices are a guess or are obtained averaging rates from previous studies. In these situations, a population matrix is available but the numbers of individuals used to

construct the matrix is unknown. There is nothing wrong with using this kind of matrices. However, to compare populations and to construct confidence intervals, a number of individuals and their class distribution must be given to the program (see below).

With matrix data, anonymous reproduction must be assumed. Recruits are allocated identically to all individuals within the same reproductive class (i.e. all individuals are assumed to have been reproductive). The format of the input data depends on the nature of the census (pre- or post-breeding) and whether they are *observed data* or are *simulated number of individuals*.

1.5.1 Pre-breeding census

For observed data, input files, read from left to right, have this structure: [Fecundity matrix] [Transition matrix] [Population vector].

Example for a population with 3 classes:

[Fecundity]			[Transition]			[N]
0	2	3	0	0	0	45
0	0	0	0.5	0.2	0	50
0	0	0	0	0.3	0.5	52

The file with input data cannot have labels or empty rows or columns (see also section **1.6** for formats):

```
0 2 3 0   0   0   45
0 0 0 0.5 0.2 0   50
0 0 0 0   0.3 0.5 52
```

If the number of individuals from which the matrix was obtained and the population structure is not known (only the matrix is available), activate the *Simulated number of individuals* option. However, because the statistical inference needs the number of individuals used to construct the matrix, a number of individuals must be given to the program. That number can be an approximate, hypothetical or tentative number of individuals simulating a study from which the matrix was obtained. The last column of the input file contains, in the first element, the total number of hypothetical individuals to be evaluated. The program will adjust the population structure to the stable stage structure. [The program only reads the first element in the N column, if individuals are allocated to other classes those will be ignored].

For example, to test the matrix assuming 147 individuals in the population:

[Fecundity]			[Transition]			[N]
0	2	3	0	0	0	147
0	0	0	0.5	0.2	0	0
0	0	0	0	0.3	0.5	0

1.5.2 Post-breeding census

The user must identify the reproductive classes, as they might not be evident from the fecundity matrix. The input file has an additional column that indicates the reproductive classes (1 or 0). In the example below, only the 2nd and the 3rd classes are reproductive:

[Fecundity]			[Transition]			[N]	[R]
0.8	2	3	0	0	0	45	0
0	0	0	0.5	0.2	0	50	1
0	0	0	0	0.3	0.5	52	1

This example is for real data. For simulated data, numbers in the N column are as above.

1.5.3 Test for matrix entries

This an advanced feature. Be sure you completely understand what you are doing at every step. You need to know how the projection matrix is constructed from the vital rates.

When input data come as a matrix (in any form: projection matrix, fecundity + transition...) there are two potential sources of error in the construction and subsequent evaluation of confidence intervals for the matrix:

The first is related to the erroneous calculation of fecundities (e.g. poor understanding of the differences between pre- and post-breeding censuses or between fecundity and fertility). In post-breeding censuses, these mistakes may be reflected as inconsistencies between the values of the transitions and the fecundities. The post-breeding matrix itself has all the information to evaluate if the calculations were correct. As an

example, we assume that the following matrix came from a post-breeding census:

$$\begin{pmatrix} 0 & 2 & 4 \\ 0.2 & 0 & 0 \\ 0 & 0.5 & 0.6 \end{pmatrix}$$

The matrix is erroneous. The pattern of transitions cannot generate the observed fecundities. In a post-breeding census, the fecundity is a composite vital rate. It is the combination of fertility and survival of reproductive individuals. In the above matrix, we must assume that only the third class is reproductive with fertility *m*.

Fecundity of class 2 = Survival of class 2 x *m:*

Fecundity of class 3 = Survival of class 3 x *m*

That is: $2 = 0.5 \times m$

$$4 = 0.6 \times m$$

There is not solution for *m*.

On finding the inconsistence, the program tries to estimate the correct fecundities for some of the classes (see next section, *1.5.4*). Very often, the result is far from being precise. So, be very careful if the problem appears and the deviation is large. The problem is even worse if the next error also occurs.

The second source of error is related to the numbers of individuals studied. To obtain the auxiliary matrix, the program needs the number of individuals used to construct the matrix. If this number is not available, or the matrix was constructed collating vital rates from different studies and populations, a realistic number must be given; and this number may cause the problem. Unrealistic or biased confidence intervals are likely when obtaining bootstrap matrices if the number is not known. The problem also appears if we know the number but the matrix entries were rounded.

An example will illustrate the problem. Let us consider the same matrix as above. This matrix has a lambda of 1.0974. And let's consider that was obtained studying 29 individuals in class 1, 57 of class 2 and 61 of class 3. But with these numbers, the matrix should be (to 4 decimal places):

$$\begin{pmatrix} 0.0000 & 2.0351 & 4.0437 \\ 0.2069 & 0.0000 & 0.0000 \\ 0.0000 & 0.5088 & 0.6066 \end{pmatrix}$$

The difference does not seem relevant but now lambda = 1.1189. The program obtains the intervals for the second, corrected, matrix (supposed to be the real matrix), not from the input matrix.

Clicking on the *Test for matrix entries* will do both analyses if census is post-breeding, or only the density test in matrices coming from pre-breeding censuses.

All the outputs below are for the same matrix as above [and with numbers of individuals of 29, 57 and 61 for classes 1 to 3. Lambda = 1.0974].

<u>Test for fecundities</u>

The test for fecundity is done with matrices corrected for density. In the example below, there is a difference between the two matrices before and after the fecundity adjustment. None of them is the input matrix.

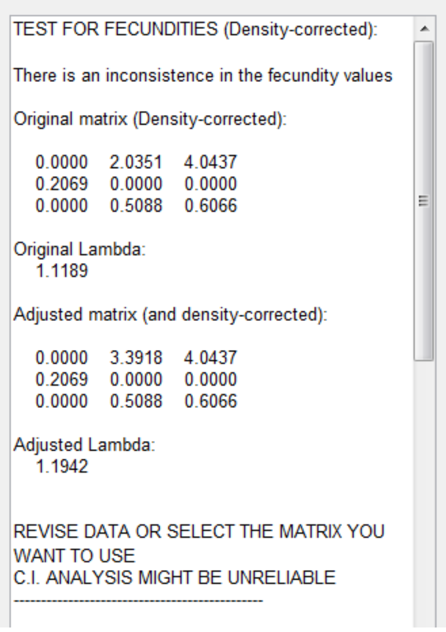
TEST FOR FECUNDITIES (Density-corrected):

There is an inconsistence in the fecundity values

Original matrix (Density-corrected):

0.0000	2.0351	4.0437
0.2069	0.0000	0.0000
0.0000	0.5088	0.6066

Original Lambda:
1.1189

Adjusted matrix (and density-corrected):

0.0000	3.3918	4.0437
0.2069	0.0000	0.0000
0.0000	0.5088	0.6066

Adjusted Lambda:
1.1942

REVISE DATA OR SELECT THE MATRIX YOU WANT TO USE
C.I. ANALYSIS MIGHT BE UNRELIABLE

Tests for densities

Two density tests are made by the program. For the first test, no fecundity adjustment is done. The first matrix is the input matrix, without any correction. The second matrix is density-corrected but not fecundity-adjusted:

```
TESTS FOR DENSITIES AND MATRIX ENTRIES:

FIRST FOR NOT FECUNDITY-ADJUSTED
MATRICES:

There is incongruence in the matrix entries

Original matrix:

   0.0000   2.0000   4.0000
   0.2000   0.0000   0.0000
   0.0000   0.5000   0.6000

Original Lambda:
   1.0974

Matrix after density correction:

   0.0000   2.0351   4.0437
   0.2069   0.0000   0.0000
   0.0000   0.5088   0.6066

Density-corrected Lambda:
   1.1189


REVISE DATA AND/OR DENSITIES

RESULTS MIGHT BE UNRELIABLE
C.I ANALYSIS IS DONE ON THE
DENSITY-CORRECTED MATRIX
```

For the second test, the fecundity is adjusted:

```
NOW, FOR FECUNDITY-ADJUSTED MATRICES:

There is incongruence in the matrix entries

Original matrix:

   0.0000   3.3333   4.0000
   0.2000   0.0000   0.0000
   0.0000   0.5000   0.6000

Original Lambda:
   1.1699

Density- and Fecundity-adjusted Matrix:

   0.0000   3.3918   4.0437
   0.2069   0.0000   0.0000
   0.0000   0.5088   0.6066

Density- and Fecundity-adjusted Lambda:
   1.1942


REVISE DATA AND/OR DENSITIES

RESULTS MIGHT BE UNRELIABLE
C.I ANALYSIS IS DONE ON THE
DENSITY-CORRECTED MATRIX
```

The first matrix is adjusted for fecundity but not density correcetd. The second matrix has both corrections.

If the census was pre-breeding, only the test for density is possible, and only for not fecundity adjusted matrices.

1.5.4 Use specific or averaged fertilities

For post breeding censuses, the fecundity matrix (***F***) is obtained from a transitions matrix (***T***) and a fertility or maternity matrix (***M***) (average number of recruits left per reproductive individual): $\boldsymbol{F} = \boldsymbol{M} \ x\ \boldsymbol{T}$. Individuals first survive and grow with probabilities given in ***T*** and then reproduce with maternities given in ***M***.

The fecundity and transitions matrices are given as input data and therefore, the maternity matrix may be obtained from $\boldsymbol{M} = \boldsymbol{F} \ x\ \boldsymbol{T}^+$, where $\boldsymbol{T}^+$ is the pseudoinverse of ***T***. When inconsistences exist in fecundities, the ***M*** matrix is an approximate solution: the best approach to the fertility values for those specific matrices of transitions and fecundity, but keeping transitions constant.

By default, the program uses the fertilities in the maternity matrix (the *specific fertility* option is selected). If inconsistences exist, a new fecundity matrix is then obtained as $\boldsymbol{F}_{new} = \boldsymbol{M} \ x\ \boldsymbol{T}$, and the new projection matrix is $\boldsymbol{A} = \boldsymbol{F}_{new} + \boldsymbol{T}$. If inconsistences are small, ***F*** and $\boldsymbol{F}_{new}$ are not very different and using ***M*** and $\boldsymbol{F}_{new}$ does not introduce large distortions in calculations. If differences are large, a completely different transition matrix appears and results may be unreliable.

There may be good reasons to stick to the original matrix even though inconsistences are severe. For example, when the matrix was obtained averaging vital rates from different areas or studies. The original matrix may be still used by following a different approach to estimate fertilities (select the *Use average fertility* option). The modification consists in that for classes with an associated fecundity, every reproductive individual reproduces with an average fertility, specific for the class, irrespective of the

destination of the individual (staying or growing). The new average fertility is obtained by dividing the original fecundity of the class by the summation of the probabilities of ending in a reproductive class. In this way, the number of recruits produced by the class is identical to the expected by the original fecundity matrix, but every reproductive individual leaves the same number of recruits irrespective of their destination.

1.6 Format for files with data

Only text files are allowed. The file must contain only numbers. Names or labels must be avoided. Data corresponding to each population are stacked in the file, with no empty rows or lines between the populations.

To compare 3 populations using raw data, anonymous reproduction of type 3, and post-breeding census, the file with data might look as:

0	0	0	[......Start of population 1]
10	15	0	
0	15	12	
30	15	12	
100	0	0	
0	1	2	
0	0	0	
4	5	0	
0	9	7	[........End of population 1]
0	0	0	[........Start of population 2]
12	18	0	
0	15	16	
35	22	15	
90	10	0	
0	1	2	
0	0	0	
3	5	0	
0	9	7	[........End of population 2]
0	0	0	[........Start of population 3]
10	15	0	
0	10	20	
40	10	10	
40	60	0	
0	1	2	
0	0	0	
4	5	0	
0	9	7	[........End of population 3]

To compare 3 populations using projection matrices with 4 classes, observed data and post-breeding census:

0.03	0.51	0.97	1.60	0	0	0	0	204	0	[....Pop 1]
0	0	0	0	0.15	0.33	0	0	145	1	
0	0	0	0	0	0.57	0.71	0	82	1	
0	0	0	0	0	0	0.23	0.92	14	1	[....End 1]
0.02	0.57	1.22	2.18	0	0	0	0	221	0	[....Pop 2]
0	0	0	0	0.10	0.51	0	0	109	1	
0	0	0	0	0	0.41	0.65	0	74	1	
0	0	0	0	0	0	0.23	0.94	18	1	[....End 2]
0.02	0.34	0.93	1.40	0	0	0	0	120	0	[....Pop 3]
0	0	0	0	0.10	0.49	0	0	118	1	
0	0	0	0	0	0.32	0.68	0	87	1	
0	0	0	0	0	0	0.24	0.83	24	1	[....End 3]

1.6.1 Limitations in the structure of the data

Limitations are consequence of simplification of the code. All populations share a common structure. They must have: (i) the same number of classes; (ii) the same type of input data; (iii) the same number of individual histories (if raw data with identified reproductives). The type of census and the sampling scheme is also common for all populations.

1.7 Load data from file

Click on this button to load population data from a text file. By default the *Data* folder in the working directory is searched. Data may also be loaded from the *File* menu or using the toolbar. Loaded data may be visualized or changed by clicking on the *Enter/Change Data* button (see below).

1.8 Enter / Change Data

Different templates are available for each of the types of input data. If data were already loaded, they are presented in the templates. They can be modified at any time. Data are saved in a temporary file as they are entered. Common options to all data types are:

Number of classes. Sets the number of classes in all populations. If data were already loaded, they are eliminated and empty templates appear.

List of populations. Shows the selected population. On selecting any population, data from the previous population are saved in a temporary file.

Done. Saves the demographic information for the populations and closes the temporary file and the window.

Save as text file. Saves the demographic data in an only text file that can be used in subsequent analyses.

Other buttons are self explanatory.

1.8.1 Input data as projection matrix

Three templates are available: One for the fecundity matrix, one for the transitions matrix and the third to enter the number of individuals studied in each class and (for post-breeding censuses) the reproductive classes. Note that the program does not evaluate the correctness of the introduced data. For the meaning of fecundity and transitions matrices, see any elementary population biology textbook (for example, Vandermeer & Goldberg, 2003).

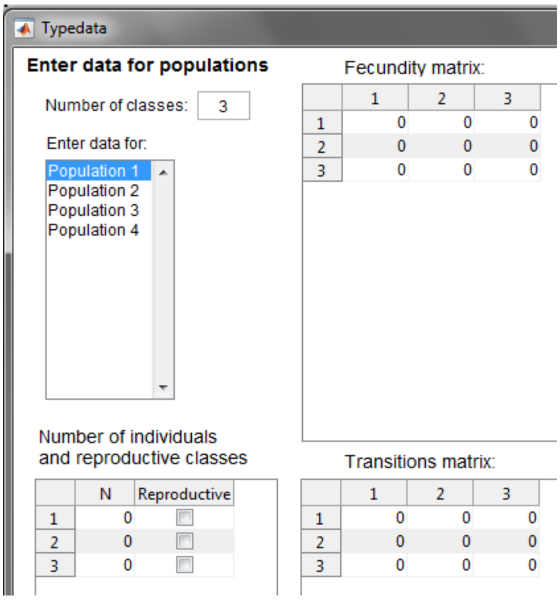

Elements of the transitions matrix should be only proportions and columns could not sum up to >1. A warning is issued is these limitations are violated. The assumption may be relaxed by deactivating the check box *Transitions are proportions only*.

1.8.2 Raw data with anonymous reproduction

One template for each population is available. Depending on the type of data, the number of rows and their meaning differs (see section **1.3**). Except for the *Relative Fertility* row, all other entries in the template are integers, as they reflect number of individuals of different type.

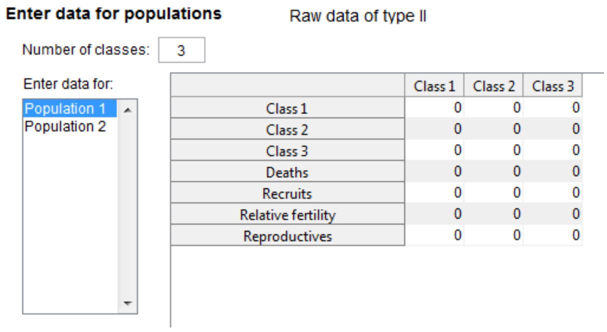

1.8.3 Raw data. Reproductives are identified

Two new options appear: the number of individual histories in the poulations and the number of classes of recruits. The number of histories refers to the total number of different histories considering all populations. If a particular history is not present in a given population, it has to be typed but it is given an abundance of 0 individuals.

The number of classes of recruits is the maximum number observed in the studied populations. If one class of recruits is never observed in a given population, the corresponding row will contain only zeros.

Alll entries in the template should be integers. They identify classes, number of recruits or the number of individuals with a particular history. Remember that individuals dying during the projection interval are given a destination class identified as the number of classes in the population +1.

There is no need for typing all histories for every population. By typing a new history in one population, that is included in all other populations with an initial density of 0 individuals. After including histories in one of the populations, for subsequent populations, only abundances need to be typed.

1.9 Adjust specifications of input data

After data have been loaded (from a file or keyboard), specifications of the type of data may still be modified on the main screen. This may lead to inconsistences with the data already loaded and may be responsible for an almost sure crash during runtime. It can also be a problem if data need to be modified or seen. A warning is issued whenever the specifications in the *Input data* panel do not coincide with the nature of the loaded data:

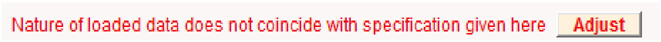

Click on the *Adjust* button to reset the specifications of the *Input data* panel, so they match the nature of the loaded data. This action does not modify loaded data.

1.10 Clear Data

Eliminates current data and results. Specifications in the main screen for input data and siumlation details remain unchanged. Data may also be eliminated selecting the option from the *File* menu or from the toolbar.

1.11 Save results

There are three ways to save the results of the analyses: (i) specifying a destination file to save the complete output during runtime, (ii) saving the content of the notification window or, (iii) to paste part or the complete output in an open document. Select the *Save results* option if you want the result be saved in a file during runtime. Before the simulations start, you will be asked for a name for the results file. By default, the *Results* folder is opened. Only numerical outputs are saved.

If the option is not selected, the results of the simulations may be still be saved; the results can still be displayed through the *Output* panel (the notification window). The option *Save text window* under the *File* menu creates a text only (*.txt*) file with the content of the notification window, whichever it is. The text window is editable. If you make annotations, these are saved with the original text. You may give details on the simulation, or eliminate parts of the information you are not interested in.

1.11.1 Graphs (Save and Print)

The graphs shown in the main screen may be send to the printer or saved anytime. Use the options under the *File* menu. When sending the graphs to the printer, some change in scale may occur. The printing options are limited to those available in the printing window of your system. The application does not offer any option for printing.

Important. Graphs are saved with a *.png* format. If the application runs as standalone, graphs always show some change in scale and aspect in relation to the graphs appearing in the main screen. If the application runs in Matlab, graphs keep their aspect only if the *export_fig* Matlab function is loaded in the Matlab path of the user's computer. Visit www.mathworks.com to download *export_fig.m* (by Yair Altman) and accompanying files and follow instructions there.

1.12 Manage folders

Destination folder to open data and save outputs may be changed any time. Click on the *Change* button or overwrite the complete directory in the edit window. If the destination folder has not the complete set of folders and sub-folders, they are created by the application.

2. ANALYSES AND SIMULATION DETAILS

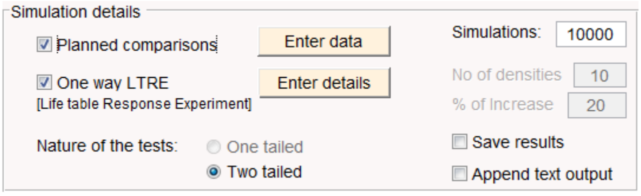

2.1 Descriptive information on populations

Descriptive information for each population include six demographic parameters that will be used to compare populations, plus sensitivity and elasticity matrices (see definitions in Caswell, 2001):

(i) The asymptotic population growth rate λ (or lambda) and the bias corrected 90 and 95% confidence intervals. The correction of bias is only relevant for large differences between the observed λ and the median obtained by simulation (e.g. differences in the second decimal place). Very often, the error associated to the estimation of λ is far larger than the calculated bias.

(ii) The net reproductive rate (R_0), defined as the average number of new individuals produced by a newly born individuals during its lifetime.

(iii) The generation time (T), estimated as the time needed by the population to increase by a factor of R_0. Because of the relationship $\lambda^T = R_0$, the generation time can be estimated as $T = lnR_0/ln\lambda$.

(iv) The stable stage structure (*SSS*), expressed as the proportion of each class in the population. If sampling was at random, the observed stage distribution (also expressed as proportion for each class) is also given. The distance between the observed and the stable stage distributions is calculated using the Keyfitz's distance. The statistical significance of the distance to SSS is obtained by a randomization test. If the number of individuals studied in each class is in some way controlled by the experimenter by setting minimum, maximum or fixed numbers of individuals, the distance to SSS and its significance are meaningless quantities and are not calculated.

(v) The reproductive value (*RV*), or contribution of each class to any other, is also expressed as the proportional contribution of each class to the abundance of all classes.

(vi) The collection of individual histories. An individual history is a summary of the events of the life cycle associated to the individuals in the population. The individual history describes the pattern of survival, growth and reproduction affecting each individual. The history specifies whether the individual survives or not and the destination class if it does; gives the number of recruits produced (in log2 classes) during the projection interval and the number of classes to which new individual recruit.

Except for the composition of histories, for each parameter, the application calculates the observed value, the 90 and 95% confidence intervals and the simulated median.

2.2 Comparing populations

Tests include global and pair-wise comparisons. Tests evaluate the null hypothesis that all populations in the study come from a single common population. The observed differences among populations should be due to sampling error associated to the random selection of individuals (with their associated histories). A significant global test indicates that at least one of the populations differ from the other as the observed differences are beyond the expected random allocation of individuals to populations. A significant global test does not identify the population(s) differing. The rationale beyond pair-wise comparisons is the same, but in this case, a significant test means that individuals from those specific populations do not have a common origin.

Test statistics differ depending on the type of test (global or pair-wise) and on the nature of the demographic parameter (scalar or vectorial). Scalar parameters are the asymptotic growth rate, the net reproductive rate and the generation time. The stable stage distribution, the reproductive value and the collection of individual histories are vectorial parameters. The three vectors are probability vectors (summing up to 1). The four test statistics below are different ways to express differences among a set of values.

For scalar population parameters in pair-wise comparisons, the test statistic is the absolute difference between the two magnitudes,

$$d_w = |x_1 - x_2|$$

where x_1 and x_2 are the parameter values for populations 1 and 2 respectively.

For global comparisons, the test statistic is the sum of squares of the parameter,

$$d_g = \sum_{i=1}^{n} (x_i - \bar{x})^2$$

where n is the number of populations, x_i is the parameter value population i and $\bar{x}$ is the average parameter across populations.

For vectorial parameters in pair-wise comparisons the test statistic is

$$D_w = \frac{1}{2} \sum_{j=1}^{m} |p_{1j} - p_{2j}|$$

where m is the number of classes if SSS or Rv are being compared among populations or the number of distinct individual histories, p_{1j} and p_{2j} are the proportions for class or history j in populations *1* or *2*. D_w is a standard measure of the difference of two probability vectors and also is the Keyfitz's Delta. D_w takes values between 0, when both vectors are identical, and 1, when no overlap exists among the two vectors.

For vectorial parameters and global comparisons the test statistic is

$$D_g = \frac{1}{2(n-1)} \sum_{j=1}^{m} \sum_{i=1}^{n} |p_{ij} - \bar{p}_{\cdot j}|$$

where m and n are as above, p_{ij} is the proportion of class or history j in population i and $\bar{p}_{\cdot j}$ is the mean proportion of class or history j across populations. *Dg* is an estimation of average distance between individual populations and the mean parameter calculated across populations. *Dw* and *Dg* are arithmetically equivalent when the number of populations is 2. *Dg* takes values between 0 and 1.

2.3 Planned comparisons

Sometimes the hypothesis of interest may be related to the differences among groups of populations. Relevant tests might be the comparison of a population against all other together (e.g. one control population against populations under different treatments) or the comparison of groups of populations sharing some common property (e.g. comparison of two or more sets of populations studied at different altitudes). Planned comparisons among *a priori* defined groups of populations are possible. To create groups of populations, all individuals from populations being grouped collapse into a single group. New demographic parameters are extracted from this new and larger population. No averaging of parameters from individual populations is done. Any number of comparisons among groups is possible: comparisons may be orthogonal when each single population or group of populations is used only once in the comparisons or non-orthogonal [but see Underwood (1997) for a discussion on the interpretation of orthogonal and non-orthogonal contrasts].

A large number of pair-wise comparisons have an associated increase in the overall probability of type I error, i.e. rejecting a true null hypothesis. Since corrections usually come at the

cost of decreased power, no action is taken by the application to this respect. The user must be aware of the problem and interpret multiple tests with caution. If needed, appropriate corrections of p-values are available (e.g. Quinn & Keough, 2002).

The option of *Planned comparisons* is active only when differences among populations are being analyzed. To define sets of contrasts and groups of populations click on the *Enter data* button. A new window opens:

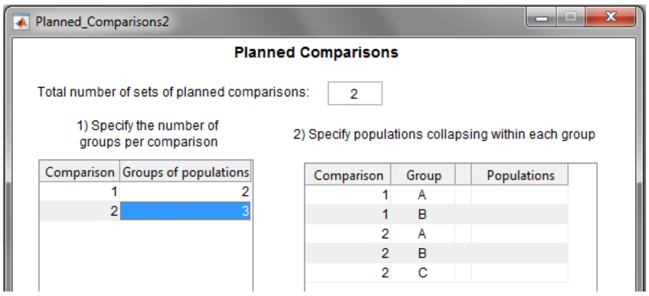

First, type the number of sets of planned comparisons in the *Total number of planned comparisons* edit window (default is 1, overwrite), then specify the groups for the planned contrasts in the two tables:

2.3.1 Specify the number of groups per comparison

It refers to the total number of groups of populations and single populations. In the example below, there are two sets of planned comparisons. The first comparison involves two groups of populations. The second comparison involves three groups. The minimum number of groups is 2 (to compare, you need at least two things).

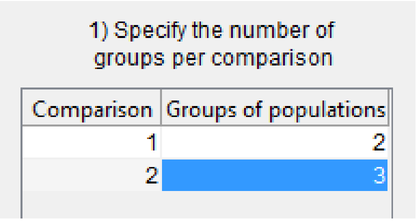

2.3.2 Specify populations collapsing within each group

In the *Populations* column, type the populations which will be collapsed to form a single unit. A group may be formed by a single population. For example, when comparing a single control with several treatments.

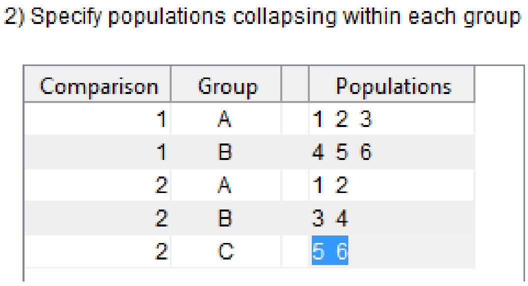

In the figure above, there are two sets of planned comparisons. Two groups of populations will be compared in the first set and three in the second. The first set compares populations 1, 2 and 3 (group A) with populations 4, 5 and 6 (group B). [So, the planned comparison consisted in the test: populations 1+2+3 *vs*. populations 4+5+6].

The second set involves 3 groups of populations: populations 1 and 2; 3 and 4; and populations 5 and 6. [So, the planned comparison consisted in the test: populations 1+2 *vs*. populations 3+4 *vs*. populations 5+6].

After entering the last group, click on the *Save and close* button. If some data need to be modified, this can be done at any time. The new data will overwrite the old ones. Closing the window or clicking on the *Cancel* button does not save any information.

2.4 Life Table Response Experiments (LTRE)

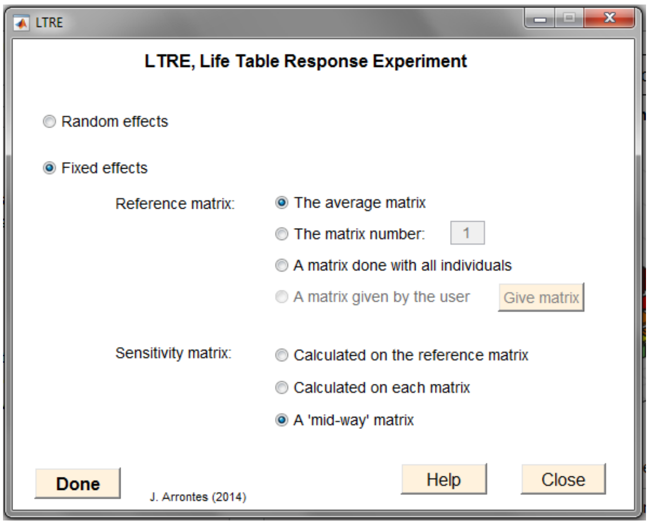

Life table Response Experiments (LTRE) are a type of perturbation analysis: It has been called *retrospective perturbation analysis*. When several populations have been studied, a range of values for λ is obtained. LTRE allow for the identification and cuantification of the vital rates responsible for the observed differences in λ. The contribution of a specific vital rate to differences in λ is related to the sesnitivity of λ to changes in the vital rate and the observed changes in the vital rate. Only one-way LTRE are available in the application. [All the information in this section, including equations, has been extracted from Caswell (2001). See additional references there].

There are two main types of LTRE depending on the nature of the factor analyzed: random and fixed effects. In short, random designs involve a sample of treatments from a hypothetically very large number of possible treatments (for example, populations studied at several randomly chosen sites). By themselves, specific treatments under a random factor have no interest because they should be selected at random. A fixed design involves treatments which are of interest by themselves. The treatments applied under fixed factors are all the possible treatments (for example, populations at the edge and at the centre of the species distribution). See any elementary statistics textbook (e.g. Quinn & Keough, 2002) for a formal definition and implications of the nature of fixed and random factors.

2.4.1 Random effects

We are not interested in changes in λ under specific treatments because they are selected at random. They do not have any specific interest by themselves. Rather, we focus on the variability in λ. For random effects designs, we analyse the contribution of each vital rate to the variance in λ*: V(λ)*

$$V(\lambda) = \sum_{ij}\sum_{kl} Cov(a_{ij}, a_{kl}) \frac{\partial \lambda}{\partial a_{ij}} \frac{\partial \lambda}{\partial a_{kl}}$$

Each element in the double summation is the contribution of a variance (when *ij* = *kl*), or a covariance among two vital rates, to the variance in lambda. It can be positive or negative (negative covariances). The partial derivatives are sensitivities of λ to changes in the vital rates.

The output of a LTRE for a random factor is a matrix in which the main diagonal are the contributions of the variances of each vital rate and the remaining elements are contributions of covariances between vital rates (of course, they appear in duplicate). The dimension of the matrix with contributions is the squared dimension of the original projection matrix. Each row and column refers to one of the elements in the population matrix.

No additional information is needed for random effects.

2.4.2 Fixed effects

The output of a LTRE for a fixed factor is a matrix for each of the populations in the study. Each matrix has the contributions of each vital rate of one population to the difference between the observed λ for that environment and a reference lambda. The value of λ in each population can be split into 2 terms: an average or reference λ and the summation of the contributions to λ of the vital rates in that specific population. Formally:

$$\lambda^{(m)} \approx \lambda^{(r)} + \sum_{ij} (a_{ij}^{m} - a_{ij}^{r}) \left. \frac{\partial \lambda}{\partial a_{ij}^{+}} \right|_{A^{+}} \qquad m = 1 \ldots n$$

where, $\lambda^{(m)}$ is the value for λ under the *m* environment. $\lambda^{(r)}$ is the reference or average value for λ which is extracted from the reference matrix and finally,

$$(a_{ij}^{m} - a_{ij}^{r}) \left. \frac{\partial \lambda}{\partial a_{ij}^{+}} \right|_{A^{+}}$$

is the contribution of the vital rate a_{ij} in the *m* matrix to the observed change in λ. To evaluate this contribution we need the sensitivity of λ to

changes in a_{ij}. This sensitivity is evaluated on a matrix $\mathbf{A}^{+}$.

To calculate the contributions of the observed changes in each vital rate to the observed variability in λ, we need a reference matrix ($\mathbf{A}^{\mathbf{r}}$). The reference matrix may be: (i) an average matrix, (ii) the matrix for a particular environment (for example, a control) or, (iii) a matrix done with all the individuals in the study. Variations in the vital rates are referred as differences between the vital rates under each of the treatments (*i.e.* populations) and the vital rates in the reference matrix. We also refer the observed population growth rate under each treatment (for each population) $(\lambda^{(m)})$ to the growth rate obtained from the reference matrix $(\lambda^{(r)})$.

The matrix used to extract sensitivities ($\mathbf{A}^{+}$) may be: (i) the reference matrix ($\mathbf{A}^{\mathbf{r}}$), whichever it is selected, (ii) the matrix for the *m* treatment ($\mathbf{A}^{\mathbf{m}}$) or, (iii) a matrix that is mid-way from the reference matrix and the matrix obtained under the *m* treatment [($\mathbf{A}^{\mathbf{r}}$+$\mathbf{A}^{\mathbf{m}}$)/2].

2.5 Evaluation of power

Non-significant results may appear because, in fact, populations have a common origin or because power was small. Power of a statistical test is defined as the probability of rejecting a false null hypothesis, or the probability of detecting a real difference. Power is positively related to sample size, the magnitude effect and the significance level (Quinn & Keough, 2002). Sample sizes refers to the number of individuals studied.

An evaluation of power is important but seldom possible before the experiment. After the study an evaluation of power may help to understand the output. After a non-significant test, small power does not give any indirect support to our hypothesis. It only says that we had a low probability to detect a significant difference but gives some indications (in terms of number of individuals) for future studies.

The application calculates power of the tests used to compare populations, both pair-wise and global by using randomization and the bootstrap. These techniques use the information contained in the samples of individuals. To calculate power, in first instance the application obtains the distribution of the test statistic under the null hypothesis. The null hypothesis assumes that all individuals have a common origin. Individuals from the populations being compared are pooled and then individuals are randomly allocated (without replacement) to the populations and the test statistic calculated again. From the distribution of simulated values, a critical value of the test statistic (leaving out 5% of largest observations) is obtained. Now, the application obtains the distribution of the test statistic assuming that the null hypothesis is false. Populations are independently resampled with replacement (simulating a new sample from each original population) and the test statistics computed again from the bootstrap samples. The proportion of values from this distribution larger than the critical value obtained assuming a true null hypothesis is an estimation of power.

In the graph below, blue bars are the distribution of the statistic under the null hypothesis (differences among populations are consequence of the random allocation of individuals to populations). The critical value, 0.12359, leaves out the 5% of larger values to the right of the distribution. The yellow bars are the distribution of the test statistic when the null hypothesis is false (populations differ beyond sampling error). Power, 0.4091, is the proportion of observations of the test statistic larger than the critical value.

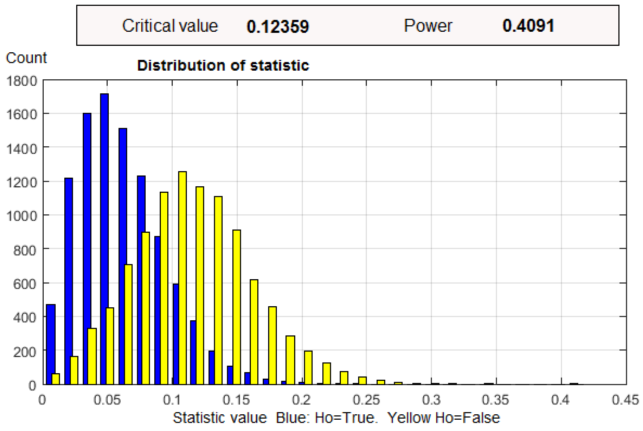

In addition to power for the observed data, the application evaluates power with increasing densities in populations; estimates the variation of the p-values of comparisons tests with increasing densities and calculates confidence intervals. In the three cases, the application evaluates *what would happen* if an increased number of individuals was sampled and identical vital rates, and hence, the same projection matrices obtained. The application assumes that the observed differences among populations are real and therefore evaluates the required increase in sample size to consider the observed differences as statistically significant. Of course, if we were to sample again the original populations with an increased effort, the new projection matrices would be expected to differ from the original ones, just by chance. But if the difference among populations was real, the new matrices would be still different Similarly, if the populations had in fact a common origin, the new matrices should be expected to be more similar than they were in the original data set and increase densities would not lead to significant tests.

In other words, the application identifies which sampling size is needed to detect if the observed differences among population are or not beyond sampling error. This is may be interpreted as an indirect estimation of power. If large increments in density are necessary to obtain significant tests, the conclusion is that a lack of power is not responsible for the results. If modest increases in density lead to significant tests, lack of power must be considered. It does not suggests that differences among populations exist.

2.6 Additional details

2.6.1 Number of simulations

This is he number of bootstrap samples and number of randomizations performed. The default number (10000) may be considered an adequate number. Overwrite the default number. The number of simulations is used for the obtention of confidence intervals, for comparisons of populations and for the evaluation of power.

2.6.2 Nature of the tests

At present, only two tailed tests are available. This might be the most common, as the direction of the difference among populations not always can be advanced.

2.6.3 Number of densities and % of increase

This option is only available when the *Evaluate Power* option is selected. This is the number of extra densities used to construct confidence intervals and to obtain differences among populations for an indirect evaluation of power. The number of individuals in each class is rounded to the nearest integer.

% of increase refers to the increase in the number of individuals from a density to the next. The increase in the population size is done proportional to the distribution of individuals into classes given in the data file.

2.6.4 Run the application and clear data

-To run the application, select the *Calculate* option from the *Analysis* menu, click on the *Calculate* button, use the combination of keys *Ctrl+R* or use the toolbar:

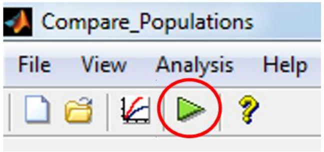

-*Clear data* eliminates all data in memory (populations data and results) but keeps the view of the screen. Select the option in the *File* menu, select it from the toolbar or click on the *Clear data* button:

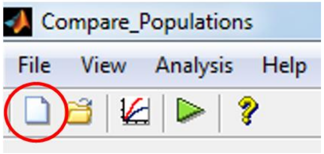

-Running a simulation eliminates the output from the last simulation.

2.6.5 Append text output

By default, when results appear in the text window, previous outputs are eliminated. If there is an interest in keeping results of previous analyses, activate this option. This may result in a very long text. To eliminate the content of the text window, click on the *Clear text* button. The cleared text cannot be recovered.

3. OUTPUT

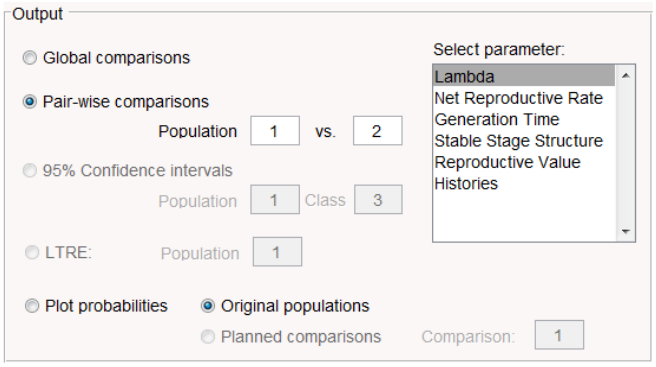

Two types of results are given by the application: numerical results and graphs. Graphs have a mere illustrative purpose and are not included in the saved outputs. Numerical results include confidence intervals and significance of comparisons. Numerical results can be inspected in the results window and may be saved to a *text* file if the option *Save results* was selected. After a simulation is run, the distribution of pair-wise differences in demographic parameters, significance levels and confidence intervals may be recovered any time and shown on the screen. Any change on the conditions of the simulations do not affect the result of the last simulation. Results may be saved at any time by saving the text window (*Save text window*, within the *File* menu). Graphs may also be saved and printed (*File* menu).

Presentation of numerical results and graphical outputs can be shifted any time. Select the option from the *View* menu or use the apropriate button in the toolbar:

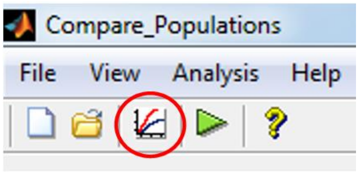

If the output in the results window are difficult to read, the window can be resized by using the *Resize window* button.

The results are kept in memory until the program is run again or data are cleared by clicking on the *Clear data* button (see above, section *2.6.4*) . Results may be authomatically saved during the simulations. If the *Save results* option is not activated when the simulation is run, results can still be saved by using the option *Save text window* under the *File* menu.

Depending on the nature of the analyses (*Evaluate Differences vs. Evaluate Power*), results and visualization options differ.

3.1 Demographic information

As basic demography for every population the program calculates:

-The population growth rate (λ), the collection of individual histories, the stable stage structure (SSS), the distance to the SSS, the reproductive value (RV), sensitivity and elasticity matrices, the net reproductive Rate (R_0) and the generation time (T).

-Also estimates medians of bootstrap values and the 95 and 90% confidence intervals for lambda, the R_0, T, the SSS and the RV.

3.1.1 The stage structure

Both the observed and the stable stage structure (SSS) are expressed as the proportion of each class in the population (and sum up to 1). The observed stage structure and the distance to the

stable structure are only given when sampling of individuals was at random and the observed structure reflects the structure of the population. If a fixed or a maximum number of individuals per class were studied, the observed stage structure is completely useless, as it is any comparison with the SSS.

The distance of the observed stage structure to the SSS is estimated as Keyfitz's distance (half of the summation of the absolute differences among the proportions of each class). To evalaute if the observed distance is statistically significant, the program uses a bootstrap procedure. By sampling a multinomial distribution with the observed proportions of each class a large number of simulated stage distributions is obtained. Simulated stage distribtions are compared with the SSS and a p-value obtained. The p-value gives the probability of obtaining a larger or identical distance by random allocation of individuals into classes.

Confidence intervals for SSS are always given and are obtained after taking bootstrap samples of individuals. New projection matrices are obtained with the new sets of individuals and the new, simulated, SSS obtained. Confidence intervals are calculated for each class.

3.1.2 Individual histories

The collection of individual histories demands a larger explanation. An individual history is a simplification of the processes affecting one individual during the projection interval: whether the individual survives or not, whether it grows, the destination class (if it does grow) and the number of recruits (new individuals incorporated into the population) produced. Individual histories are presented in a table with 5 rows and a variable number of columns. All this information can be extracted from the input matrix or from raw population data (see Section 1 in this document).

Important: The matrix with individual histories is a condensed version of the auxiliary matrix used for the bootstrap and randomization tests. It is also different to the input matrices for *identified reproductives* data. **Do not use as an input**.

Below is part of one of these tables for a population in which 3 classes or classes are identified:

```
HISTORIES:

Origin           1    1    1    1    1    2    2
Destination      2    2    2    2    4    2    2
No. Recruits     0    2    2    3    0    0    2
...in classes    0    1    2    2    0    0    1
Individuals      9    2    0    1   35   13    3
```

Each column is a different history. The first row, *Origin*, identifies the class at which the individual was at the beginning of the projection interval (at time *t*).

The second row, *Destination*, is the class at which the individual is at the end of the projection interval (at time *t+1*). If the individual dies during the projection interval, its destination class is identified as the *number of classes +1* (3+1=4 in this example).

The third row, *No. of Recruits*, is the number of new individuals added to the population by an individual. If the individual did not reproduce or all its descendants died during the projection interval, then the number of recruits left is 0. Surviving recruits are presented in the table as log_2 classes:

0: No recruits left;
1: 1 recruit produced;
2: 2 or 3 recruits produced;
3: 4,5,6 or 7 recruits produced;
4: 8-15 recruits produced, and so on.

The fourth row, *in classes*, specifies the number of classes to which new individuals recruit. In most cases recruitment is limited to the first class, but in same populations and species, fast grow may be responsible for some individuals appearing in higher classes. The number of classes is *0* if no recruits were left. If all recruits appear in the population in the same class, then it is *1*. If they appeared in 2 different classes, it is *2*, and so on. For example, if one individual left 5 recruits and, at the end of the projection interval, all of them were in the same class, then it should be *1*. If at least one of the recruits was in a different class (due to fast grow), then it should be *2*.

The last row, *Individuals*, gives the number of individuals in the population with that specific history. If the number is *0*, it means that the history does not appear in the population, but it was present in at least one of the other populations studied.

One example will help: the fourth column of the table is for individuals which were in class 1 at the beginning of the projection interval, they survived and ended in class 2, they reproduced and left 4 to 7 recruits which were in 2 different classes. There was a single individual with that particular history in the population.

3.1.3 Other parameters

The net reproductive rate (R_0) (or basic reproductive rate) is defined as the life time expected offspring production by a newly born individual. For any population, if fecundieties are reduced by a factor of $1/R_0$, then the population reaches a stationary state with $\lambda = 1$.

The generation time is the time need for the population to grow by a factor of R_0.

The reproductive value is expressed as the proportional contribution of each class to any other classes in the population. Confidence intervals are obtained as for the SSS.

3.2 Differences among populations

Comparisons of populations include global and pairwise differences among populations and their associated p-values

And, if the options were selected:

-Planned (a priori) comparisons among groups of populations.

-Life Table Response Experiment (LTRE)

All the above calculations may be saved in a results text file.

3.2.1 Global and pairwise comparisons

The output panel offers options to visualize the results of the comparisons, in the graph window and the accompanying notification area. The graph shows the distribution of the differences among populations after the randomization; the original difference and its associated p-value are shown in the notification area.

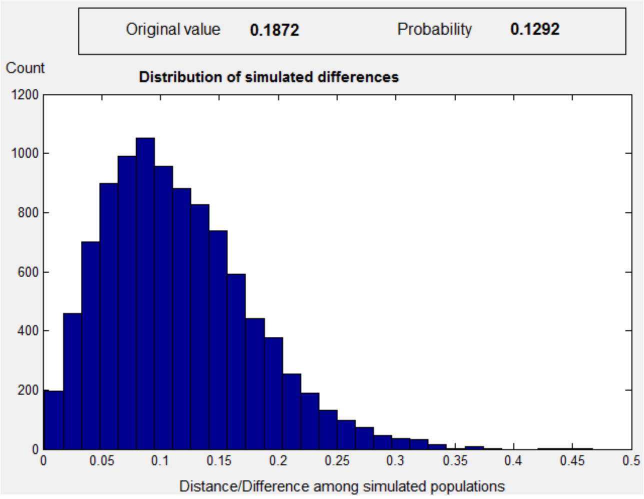

Specify whether global or pairwise comparisons, the demographic parameter being compared and, for pairwise comparisons, the populations being compared. This graphical output with the distribtion of the simulated differences is only for the original populations. For planned comparisons the information is in the text window (but see next section).

3.2.2 Ploting probabilities (and planned comparisons)

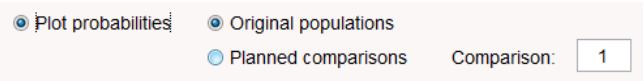

Probabilities for global and pairwise tests may be plotted for comparison. If planned comparisons were done, the p-value associated to each comparison can be also be plotted.

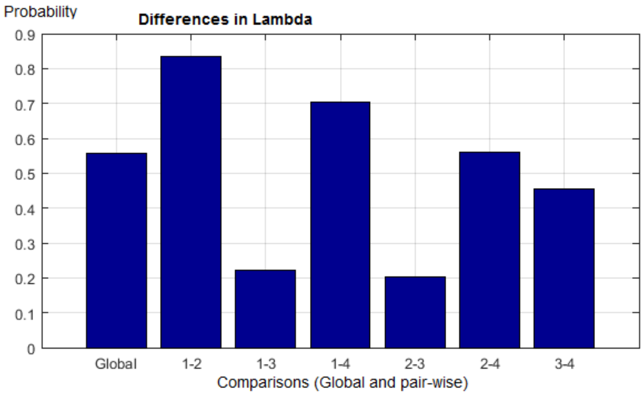

In the graph, global pand pairwise p-values are plotted for differences in lambdas among 4 populations.

If only two populations were studied, or two *a priori* groups were defined for planned comparisons, only one probability exist per demographic parameter and no result is presented. The probability can be seen in the text window.

3.2.3 Life Table Response Experiment (LTRE)

The contribution of the vital rates to lambda is displayed in a different way depending on the nature (random *vs* fixed) of the sampling design.

For random effects, the numerical output is a symmetrical matrix whose elements are the contributions of variances and covariances among vital rates to the observed variance of lambda:

```
LIFE TABLE RESPONSE EXPERIMENT (LTRE):
(Random factors)

Identification of vital rates:

   1    4    7
   2    5    8
   3    6    9

Matrix with contribution of vital rates:

          1         2         3         4
  1  0.00024 -0.00012  0.00000  0.00026 -0.(
  2 -0.00012  0.00010  0.00000 -0.00022  0.(
  3  0.00000  0.00000  0.00000  0.00000  0.(
  4  0.00026 -0.00022  0.00000  0.00048 -0.(
  5 -0.00166  0.00007  0.00000 -0.00019  0.(
  6  0.00020 -0.00012  0.00000  0.00027 -0.(
  7  0.00036 -0.00013  0.00000  0.00030 -0.(
  8 -0.00059  0.00010  0.00000 -0.00024  0.(
  9 -0.00020 -0.00001  0.00000  0.00001  0.(
```

The vital rates above are coded, from 1 to 9. They are identified by its position in the population matrix (see above). For example, for a population with three classes, the vital rate number 4 is the fecundity of class 2, the vital rate number 9 is the probability of individuals in class 3 to survive and remain.

The main diagonal of the matrix with the contributions of vital rates are the variances of the vital rates. All other elements are the covariances of each pair of vital rates. The matrix is symmetrical.

To visualice contributions in a graph, switch to the graphical output and select the *LTRE* option. A 3D graph of the matrix appears. The vertical axis (Z) are the contributions of the variances and covariances to the variance in lambda. The X and Y axes are the vital rates. The diagonal are variances. Any other element is a covariance among rates.

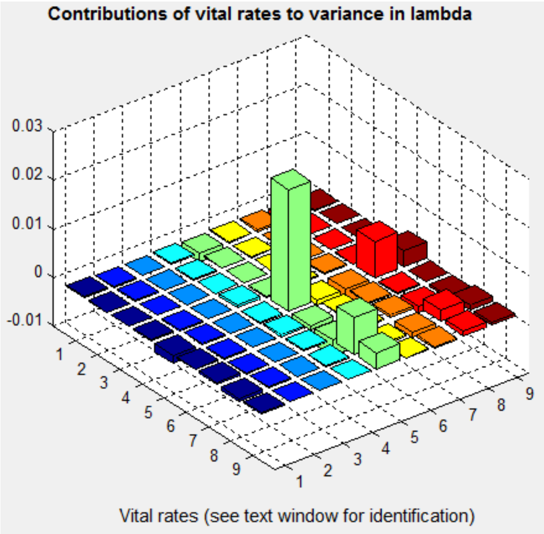

For fixed effects, the output is different:

```
LIFE TABLE RESPONSE EXPERIMENT (LTRE):
(Fixed factors)

Population no. 1:

   0.0227   0.0214   0.0304
  -0.0107  -0.0859  -0.0442
   0.0000   0.0146  -0.0154

Population no. 2:

  -0.0071  -0.0218  -0.0031
   0.0096  -0.0441  -0.0135
   0.0000  -0.0103  -0.0121

Population no. 3:

  -0.0069   0.0045  -0.0174
  -0.0016   0.2221   0.0500
   0.0000  -0.0048   0.0225
```

Contributions of vital rates are given for each population. In this case, values are the contribution of each vital rate in a given population to the difference between the lambda in that population and the lambda in a reference matrix. To visualize the data in a graph, just type the apropriate population in the edit box. Vital rates shown in the graph are coded as in the random effects case above. The numbers identify the position of the vital rate in the population matrix.

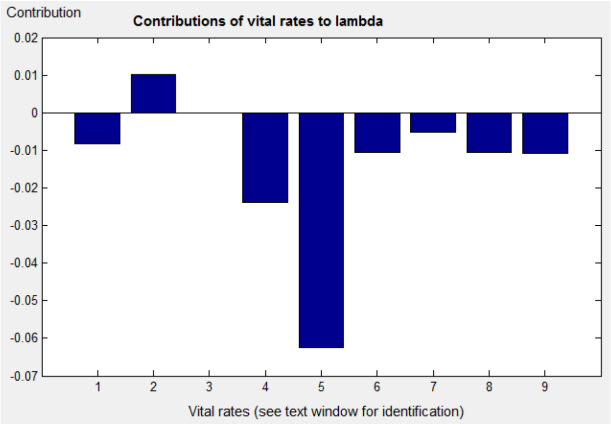

3.3 Evaluation of power

The program calculates power of global and pairwise comparisons for all six demographic parameters. Power is estimated for a significance level of 0.05 and two tailed tests. Power is estimated for the real observed data and for a range of densities given by the user, but keeping the composition of life histories constant for each population. In addition, for each density, the program calculates:

-The 90 and 95% confidence intervals for demographic parameters in each population.

-The p-values of the global differences among populations

-The p-values associated to pair-wise differences among populations in the six demographic parameters.

All the above calculations are shown in the text window and may be saved in the results text file.

No planned comparisons are yet implemented for evaluation of power and, thus, this option is disabled in the *Output* panel.

3.3.1 Global and pair-wise comparisons

To display a particular graph, select the demographic parameter from the listbox and the graph type from the pop-up menu. Three different results and graphs are available:

-Graph for power calculated with real data.
-The variation of power with densitity.
-The variation of the p-values of comparisons with increasing densities.

<u>Power graph</u>

The graph is only for real data (density 0) and shows the distribution of differences under the null hypothesis (blue bars in the graph below) and the distribution of differences when the null hypothesis if false (yellow bars). The critical value of the statistic for a significance level of 0.05 and power are above the graph.

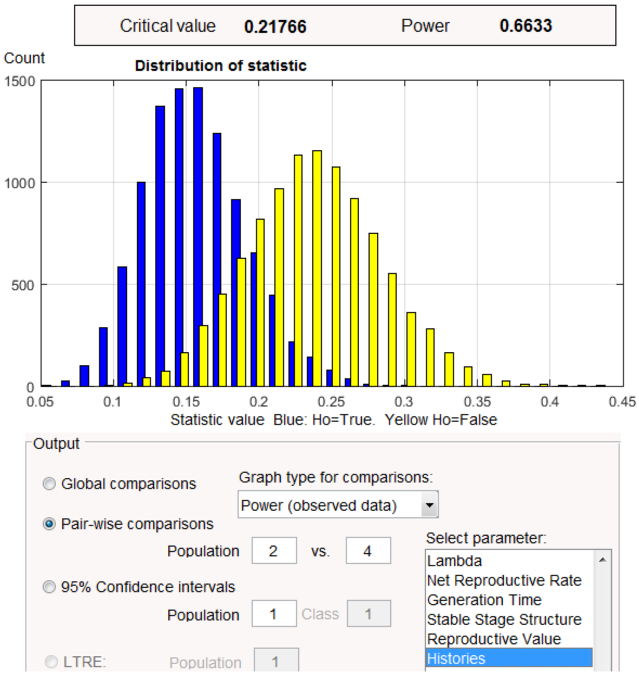

<u>Power *vs.* density</u>

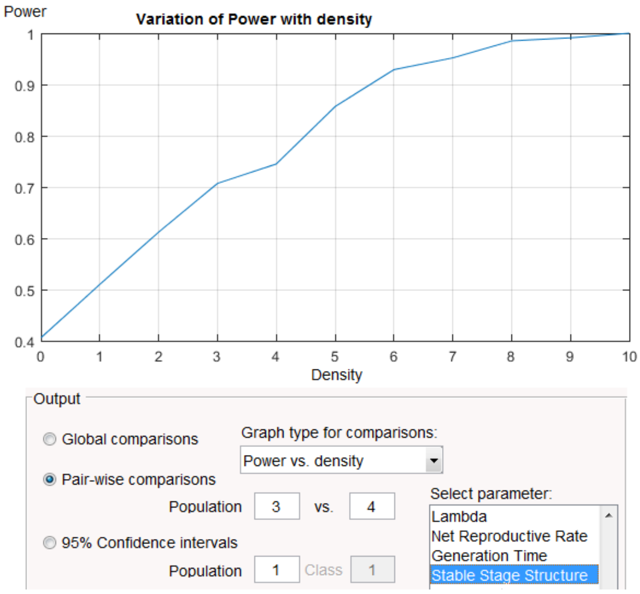

Power is simulated for increasing densities assuming constant vital rates (i.e. identical composition of life histories). The graph below shows power variation with density for the comparison of the estable stage structure of populations 3 and 4.

The codes for the densities used are defined in the text window:

Density	Pop 1	Pop 2	Pop 3	Pop 4
0	109	133	115	109
1	130	159	138	131
2	157	192	165	156
3	189	230	198	188
4	226	276	239	226
5	271	331	287	272
6	327	399	344	327
7	391	476	413	391
8	468	571	494	468
9	562	687	594	563
10	675	823	713	676

In the above example, the real densities for populations 1 to 4 were 109, 133, 115 and 109 individuals respectively (density 0). Ten additional densities were tested, with an increment of a 20% from one density to the next. Thus, the last set of densities tested were 675, 823, 713 and 676 individuals for each of the populations. Power should be around 0.85 for density 5: 287 and 272 individuals, for population 3 and 4 respectively. The interpretation is that if the vital rates in both populations remain unchanged, sampling above the 5th density should have a good chance to detect significant differences in the stable stage structure

<u>P-value *vs.* density</u>

The graph shows a simulation of how p-values of tests for comparison of populations change with density. Simulations assume, as before, that the vital rates in the populations remain constant.

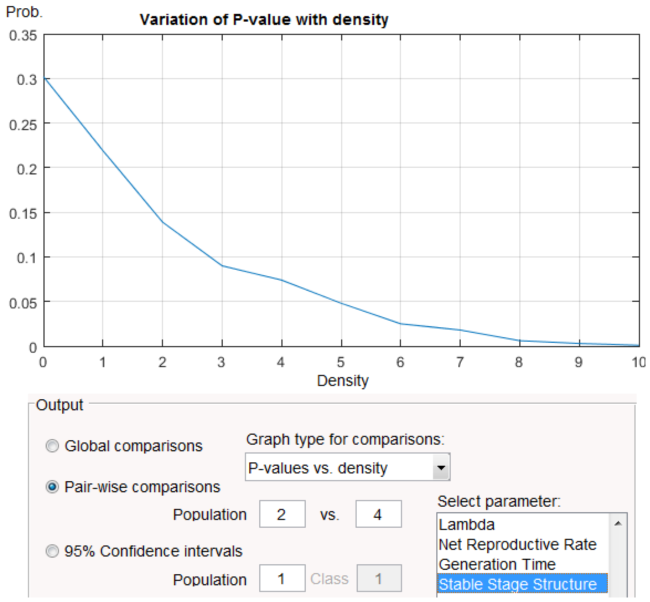

The results say (see graph) that above the 5th population sizes, the tests on differences in stable stage structure for populations 2 and 4 would render significant results. The alternative interpretation is that to detect significant differences among those populations, the study should have been done on a minimum of 331 individuals for population 2 and 272 for population 4 (instead of 133 and 109 respectively). All this assuming that the demographic data were obtained without error.

3.3.2 Confidence intervals

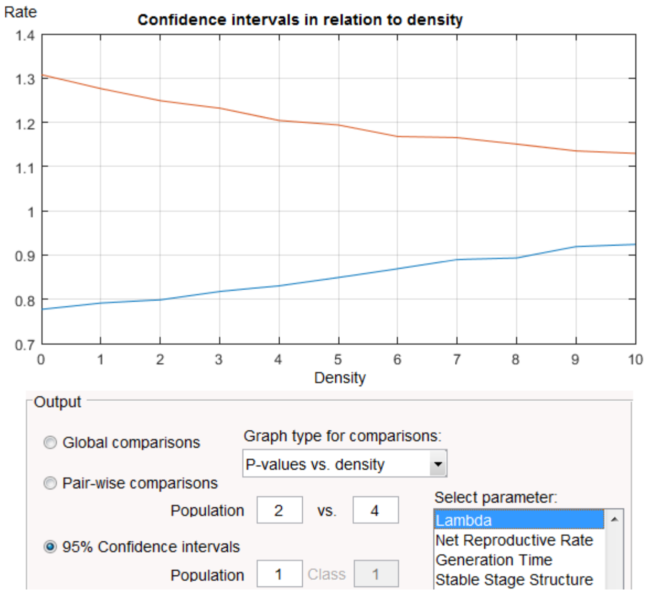

Select the demographic parameter and the population being tested. Confidence intervals are not available for *Histories*.

If the intervals are for the SSS or RVs, both the population and the class within the population must be specified.

The example above shows the variation of the confidence interval for lambda with increasing densities for population 1. Lines in the graph represent both extrems of the interval. Only the 95% CIs are displayed in the graph.

LITERATURE CITED

Caswell, H. 2001. *Matrix population models. Construction, analysis, and interpretation*. 2nd edition. Sinauer, Sunderland.

Hastings, A. 1997. *Population biology. Concepts and models*. Springer, New York.

Manly, B.F.J. 2007. *Randomization, bootstrap and Monte Carlo methods in biology*. 3rd edition. Chapman & Hall, Boca Raton.

Quinn, G.P & Keough, M.J. 2002. *Experimental design and data analysis for biologists*. Cambridge University Press, Cambridge.

Rockwood, L.L. 2006. *Introduction to population ecology*. Blackwell, Malden

Underwood, A.J. 1999. *Experiments in ecology. Their logical design and interpretation using analysis of variance*. Cambridge University Press, Cambridge

Vandermeer, J.H. & Goldberg, D.E. 2003. *Population ecology. First principles*. Princeton University Press, Princeton.

Quick reference guide for `Pop_Inference`

Options	Action
**File menu**	
Load data	Imports text file with demographic data
Save graph	Saves current graph in .png format (some change in shape/scale may occur)
Save text window	Saves content of text window in a text file
Print graph	Sends current graph to the printer using resources of the system
Print result	Not yet available
Clear data	Releases memories and eliminates current data and results (if any)
**View menu**	
Show/Hide graph and text	Switches the view of the output between graph and text window
**Analysis menu**	
Evaluate differences	Compares populations
Evaluate power	Changes in p-values and confidence intervals with increasing number of individuals
Calculate	Runs the simulations
**Help menu**	
Help	Opens a html help
Printable pdf	Opens help in a printable pdf
About	Self-acknowledgement and some disclaimer lines
**Input data**	
Random sampling	Number of individuals at each class reflects real structure in the field
Fixed number of individuals	The number of individuals per class do not reflect the true relative abundance of the class
Number of populations	Minimum 2 populations. No upper limit. Single population studies possible (descriptive)
Pre-breeding census	Individuals in populations were counted, sexed and aged before the reproductive period
Post-breeding census	Individuals in populations were counted, sexed and aged after the reproductive period
Raw data as input	Raw demographic data are used.
Anonymous reproduction	Recruits cannot be assigned to specific reproductive individuals
Type I	Reproductives cannot be identified
Type II	Reproductives identified but their origin or destination is unknown
Type III	Reproductives identified with known origin and destination
Identified reproduction	The parents for every recruit are known
Matrix as input	Demographic data given as a transition matrix
Observed data	The number of individuals per class used to construct the matrix is known and is given
Simulated no. of individuals	Unknown number of individuals used. Population is at Stable Stage Structure
Test for matrix entries	Evaluates correctness of matrix entries. Needs identification of the matrix to be evaluated
Use specific fertility	Simulations use adjusted fecundities (even after errors in the construction of the matrix)
Use average fertility	Simulations use always original fecundities, averaging fertilities within classes
Adjust	Resets specifications of input data in the main screen to match that of already loaded data
Load data from file	Loads data from a text only (*.txt*) file
Enter / Change Data	Allows for the introduction, modification and visualization of data using specific templates
Clear Data	Eliminates demographic data
**Simulation details**	
Planned comparisons	*A priori* comparisons. Compares groups of populations defined by the user. Needs details
One-way LTRE	Life table response experiments: A kind of perturbation analysis. Needs additional details
Nature of tests	Only two tailed tests available
Simulations	Number of bootstrap samples and randomizations. The bigger the number, the better
Number of densities	Specifies the number of increasing densities evaluated for Power analysis
% of increase	How densities increase in Power analysis
Save results	Saves results during simulations. A name for output file is given at runtime
Append text output	New results are appended to previous information in the text window
Calculate	Runs analysis
**Output**	
Select graph type	Self explained. For power analysis only
Select parameter	Select parameters to be graphed for p-values, differences, confidence intervals or power
Global comparisons	Shows results for global differences (comparisons or power)
Pairwise comparisons	Shows results for pairwise differences (comparisons or power) between populations
Confidence intervals	Shows changes in confidence intervals with growing numbers for demographic parameters
LTRE	Shows results from LTRE analysis
Plot probabilities	P-values for global and pairwise differences for populations or planned comparisons
Clear text	The text window is cleared. Information cannot be recovered
Resize window	Resizes text window
