# Supplementary material for: Pop‐Inference: An educational application to evaluate statistical differences among populations
Source: Ecol Evol. 2018 May 4;8(11):5224–30. doi: 10.1002/ece3.4010 (PMC6010711; doi:10.1002/ece3.4010)
Supplement: Supplementary file 2 [file ECE3-8-5224-s002.docx]

**APPENDIX S2. CONSTRUCTION OF PROJECTION MATRICES FROM RAW DATA AND OBTENTION OF AUXILIARY MATRICES FOR RANDOMIZATION AND THE BOOTSTRAP**

**Construction of projection matrices from raw data.**

Raw data always include the fates of individuals during the projection interval and varied information on reproduction. When recruits may be assigned to reproductive individuals, the input data come as a collection of individual histories. When reproduction is anonymous, there are three different formats for input data, depending on the available information. In all cases, the construction of the matrix for transitions is trivial as they are proportions of individuals with different fates (stay, grow, regress or die) obtained from the absolute numbers of individuals with each fate. The construction of the matrix for fecundities differs depending on the nature of the census (pre- or post-reproductive) and the information on reproduction.

**Anonymous reproduction Type I**

Reproductive individuals are not identified. It is assumed that every individual at the reproductive classes reproduces during the projection interval with an average fertility. See table 1 below.

Table 1. Input data with raw demographic information of type I. One hundred newborns recruited to class 1 and 25 recruited to class 2. Classes 2 and 3 were reproductive. The reproductive output of individuals at class 3 is twice the output of class 2 individuals

|  |  | Origin |  |  |
| --- | --- | --- | --- | --- |
| Destination |  | Class 1 | Class 2 | Class 3 |
|  | Class 1 | 0 | 0 | 0 |
|  | Class 2 | 10 | 15 | 0 |
|  | Class 3 | 0 | 15 | 12 |
|  | Death | 30 | 15 | 12 |
|  | Recruits | 100 | 25 | 0 |
|  | Relative Fertility | 0 | 1 | 2 |

The construction of the matrix from transitions is trivial and gives: $\left( \begin{matrix} 0 & 0 & 0 \\ 0.25 & 0.33 & 0 \\ 0 & 0.33 & 0.5 \end{matrix} \right)$.

To construct the matrix for fecundities we need the number of individuals reproducing, the total number of recruits produced during the interval and the relative fertility of individuals at different classes.

Number of individuals reproducing. The number is estimated differently if census is pre-breeding or post breeding. If it is pre-breeding, every individual at reproductive classes, and counted at time *t*, reproduces. For example, in table 1, the number of reproductives per class is: Class 1, 0; class 2, 45; class 3, 24. Because fertility of different classes may be different, we calculate the number of equivalent individuals and a gross fertility per equivalent individual (see table 2).

If more than one class of recruit exist, gross fertility is calculated for each class of recruits:

Number of recruits at class 1 produced by equivalent reproductive = 100/93

Number of recruits at class 2 produced by equivalent reproductive = 25/93

These numbers of recruits are then multiplied by the relative fertility of each class to obtain the average number of recruits produced by each reproductive individual in each class.

Table 2. Calculations to obtain the fecundity matrix in a pre-breeding census and anonymous reproduction of type I. Observed data are in normal font; estimated or derived magnitudes are in *italics*.

|  |  | Origin |  |  |
| --- | --- | --- | --- | --- |
| Destination |  | Class 1 | Class 2 | Class 3 |
|  | Class 1 | 0 | 0 | 0 |
|  | Class 2 | 10 | 15 | 0 |
|  | Class 3 | 0 | 15 | 12 |
|  | Deaths | 30 | 15 | 12 |
|  | Recruits | 100 | 25 | 0 |
|  | Relative Fertility | 0 | 1 | 2 |
|  | *Reproductives* | *0* | *45* | *24* |
|  | *Equivalent reproductive individuals* | *0* | *45* | *48* |
|  | *Average recruits at class 1 per reproductive* | *0* | *100/93* | *2*100/93* |
|  | *Average recruits at class 2 per reproductive* | *0* | *25/93* | *2*25/93* |

The matrix for fecundities is$\left( \begin{matrix} 0 & 1.075 & 2.151 \\ 0 & 0.269 & 0.538 \\ 0 & 0 & 0 \end{matrix} \right)$.

In a post-breeding census, calculations are different (table 3). After the census, individuals must survive during the projection interval to reproduce. Individuals at non-reproductive classes may have an associated fecundity, as they may become reproductive during the projection interval and reproduce with the fertility of the class they entered.

Fecundity in a post-breeding census results from pre-multiplication of a matrix of transitions by a maternity matrix, ***F*** *=* ***M*** *x* ***T***. The maternity matrix is calculated as the average number of recruits produced per reproductive individual.

Table 3. Calculations for a post-breeding census and anonymous reproduction of type I. Observed data are in normal font; estimated or derived magnitudes are in *italics*.

|  |  | Origin |  |  |
| --- | --- | --- | --- | --- |
| Destination |  | Class 1 | Class 2 | Class 3 |
|  | Class 1 | 0 | 0 | 0 |
|  | Class 2 | 10 | 15 | 0 |
|  | Class 3 | 0 | 15 | 12 |
|  | Deaths | 30 | 15 | 12 |
|  | Recruits | 100 | 25 | 0 |
|  | Relative Fertility | 0 | 1 | 2 |
|  | *Reproductives at class 2* | *10* | *15* | *0* |
|  | *Reproductives at class 3* | *0* | *15* | *12* |
|  | *Equivalent reproductive individuals at 2* | *10* | *15* | *0* |
|  | *Equivalent reproductive individuals at 3* | *0* | *30* | *24* |
|  | *Average recruits at class 1 per reproductive* | *0* | *100/79* | *2*100/79* |
|  | *Average recruits at class 2 per reproductive* | *0* | *25/79* | *2*25/79* |

The matrix for fecundities is (***F*** *=* ***M*** *x* ***T***):

$\left( \begin{matrix} 0.316 & 1.266 & 1.266 \\ 0.079 & 0.316 & 0.316 \\ 0 & 0 & 0 \end{matrix} \right)=\left( \begin{matrix} 0 & 1.266 & 2.532 \\ 0 & 0.316 & 0.633 \\ 0 & 0 & 0 \end{matrix} \right)\times\left( \begin{matrix} 0 & 0 & 0 \\ 0.25 & 0.33 & 0 \\ 0 & 0.33 & 0.5 \end{matrix} \right)$.

**Auxiliary matrix for randomization and bootstrap**

The auxiliary matrix has a number of columns identical to the number of individuals in the sample and a variable number of rows depending on the number of classes to which new individuals recruit. Each column is the history of an individual during the projection interval. Filling the two first rows is easy. The first row is the class of origin of each individual. For data in table 1, there will be, 40 columns with a 1 as first element (40 individuals started the study in class 1), 45 with a 2 and 24 with a 3. The second row is the destination class and 10 out of the 40 individuals in class 1 with have a 2 (they promote to class 2), 30 will die and will have the code for death (because the population has 3 classes, it may be 4) and so on.

Assignation of recruits to every individual is more tricky. The auxiliary matrix has an additional row for each class of recruits. In the example, the auxiliary matrix will have two additional rows. Because we do not have any information on the individuals actually reproducing, we consider that every individual at the reproductive classes reproduced. The number of recruits is allocated to every reproductive individual using integer numbers of recruits per individual. The allocation algorithm works in the same way irrespective of the nature of the census and the type of input data (matrix or raw data):

(i) Identification of the types of individuals in the population defined by origin and destination classes. In the example there are 7 types of individuals: Starting at class 1 and growing to 2, starting at 1 and dying, starting at 2 and staying in 2,…

(ii) For each type of individuals, the total number of recruits produced is calculated using the average recruit production per reproductive individual (calculated in a different way depending on the nature of input data). For example, 15 individuals starting at 2 and remaining in 2 leave an average of 1.075 recruits in class 1 and 0.269 recruits at class 2, which makes a total of 16.125 recruits in class 1 and 4.035 in class 2.

(iii) The recruits are allocated, sequentially and one by one, to all reproductive individuals.

(iv) If the total number of recruits is fractional, after the allocation of all integer recruits, the next reproductive is assigned a fraction of individual.

(v) If applicable, allocation of additional classes of recruits starts by the first reproductive individual after the last one in receiving recruits of the previous class.

The life histories for the 15 individuals should be:

$$\begin{matrix} Origin & 2 & 2 & 2 & 2 & 2 & 2 & 2 & 2 & 2 & 2 & 2 & 2 & 2 & 2 & 2 \\ Destination & 2 & 2 & 2 & 2 & 2 & 2 & 2 & 2 & 2 & 2 & 2 & 2 & 2 & 2 & 2 \\ Stage 1 recruits & 2 & 1.125 & 1 & 1 & 1 & 1 & 1 & 1 & 1 & 1 & 1 & 1 & 1 & 1 & 1 \\ Stage 2 recruits & 0 & 0 & 1 & 1 & 1 & 1 & 0.035 & 0 & 0 & 0 & 0 & 0 & 0 & 0 & 0 \end{matrix}$$

The construction of the auxiliary matrix is identical for all types of input data. The differential aspect is how the average number of recruits produced by reproductive individual is calculated for each type of individual.

**Anonymous reproduction of type II**

Not every individual in the reproductive classes reproduces. Reproductive individuals are identified but either the class of destination of the individuals (in pre-breeding censuses) or the origin (in post-breeding censuses) are not known.

In pre-breeding censuses (table 4), within each class, the destination for reproductives is allocated to the different categories depending on the probabilities in the matrix of transitions.

Table 4. Calculations for a pre-breeding census and anonymous reproduction of type II. Observed data are in normal font; estimated or derived magnitudes are in *italics*.

|  |  | Origin |  |  |
| --- | --- | --- | --- | --- |
| Destination |  | Class 1 | Class 2 | Class 3 |
|  | Class 1 | 0 | 0 | 0 |
|  | Class 2 | 10 | 15 | 0 |
|  | Class 3 | 0 | 15 | 12 |
|  | Deaths | 30 | 15 | 12 |
|  | Recruits | 100 | 0 | 0 |
|  | Relative fertility | 0 | 1 | 2 |
|  | Reproductives | 0 | 9 | 16 |
|  | *Equivalent reproductive individuals* | *0* | *9* | *32* |
|  | *Average recruits per reproductive* | *0* | *100/41* | *2*100/41* |
|  | *Probability of being reproductive* | *0* | *9/45* | *16/24* |
| *Destination for reproductives only* | |  |  |  |
|  | *Class 1* | *0* | *0* | *0* |
|  | *Class 2* | *0* | *3* | *0* |
|  | *Class 3* | *0* | *3* | *8* |
|  | *Death* | *0* | *3* | *8* |

The fecundity of each class is calculated from the average number of recruits per reproductive individual multiplied by the probability of an individual to be a reproductive within that class: $\left( \begin{matrix} 0 & 0.488 & 3.252 \\ 0 & 0 & 0 \\ 0 & 0 & 0 \end{matrix} \right)$.

The data with the destinations for reproductives are only needed for the construction of the auxiliary matrix used for randomization and the bootstrap.

In a post-breeding census (table 5), the origin of the reproductive individuals is proportionally allocated to all possible origins for a given type of reproductives. For example, reproductives at class 2 my come from individuals promoting from class 1 or from individuals remaining in class 2.

Table 5. Calculations for a post-breeding census and anonymous reproduction of type II. Observed data are in normal font; estimated or derived magnitudes are in *italics*.

|  |  | Origin |  |  |
| --- | --- | --- | --- | --- |
| Destination |  | Class 1 | Class 2 | Class 3 |
|  | Class 1 | 0 | 0 | 0 |
|  | Class 2 | 10 | 15 | 0 |
|  | Class 3 | 0 | 15 | 12 |
|  | Deaths | 30 | 15 | 12 |
|  | Recruits | 100 | 0 | 0 |
|  | Relative Fertility | 0 | 1 | 2 |
|  | Reproductives | 0 | 9 | 16 |
|  | *Equivalent reproductive individuals* | *0* | *9* | *32* |
|  | *Average recruits per reproductive* | *0* | *100/41* | *2*100/41* |
| *Reproductive individuals at t+1* | |  |  |  |
|  | *Class 1* | *0* | *0* | *0* |
|  | *Class 2* | *4* | *5* | *0* |
|  | *Class 3* | *0* | *9* | *7* |
| *Probability of becoming reproductive*  *during the projection interval* | |  |  |  |
|  | *Class 1* | *0* | *0* | *0* |
|  | *Class 2* | *4/40* | *5/45* | *0* |
|  | *Class 3* | *0* | *9/45* | *7/24* |

The matrix for fecundities (***F***) is obtained from a matrix with probabilities of individuals at each class to become reproductive (a transitions matrix for reproductives only, ***T***) pre-multiplied by the fertility matrix (***M***, the average recruits produced per reproductive individual): ***F*** = ***M*** x **T**

$\left( \begin{matrix} 0.244 & 1.246 & 1.424 \\ 0 & 0 & 0 \\ 0 & 0 & 0 \end{matrix} \right)=\left( \begin{matrix} 0 & 2.439 & 4.878 \\ 0 & 0 & 0 \\ 0 & 0 & 0 \end{matrix} \right)\times\left( \begin{matrix} 0 & 0 & 0 \\ 0.100 & 0.111 & 0 \\ 0 & 0.200 & 0.292 \end{matrix} \right)$.

**Anonymous reproduction type III**

Not every individual in the reproductive classes reproduces. Reproductives have either a known destination (in pre-breeding censuses) or a known origin (post-breeding).

The construction of the projection matrix is identical to the previous case, both for pre- and post-breeding censuses. The only difference is that numbers for origin or destinations of reproductives are a guess in type II but are the observed numbers for type III.

**Construction of the auxiliary matrix when the input data come as projection matrices**

Entering data for the two first rows in the auxiliary matrix is trivial. The matrix of transitions and the number of individuals in each class at the beginning of the projection interval give all the information. The fecundity matrix contains the average number of recruits produced per individual during the projection interval. For a pre-breeding census, every individual at reproductive classes reproduces at the beginning of the projection interval and leaves an average number of recruits specified in the fecundity matrix. The total number of recruits produced by individuals at a given class is obtained from the entry in the fecundity matrix for that class multiplied by the number of individuals in the class. Allocation of recruits to individuals in the auxiliary matrix proceeds as explained before.

In post-breeding censuses, elements in the fecundity matrix are a combination of survival and fertility. Not every individual counted at the beginning of the projection interval reproduces. Some individuals die before they reach the reproductive period at the end of the projection interval. If individuals remain in the same class, they reproduce with the fertility associated to their class. If they grow, they reproduce with the fertility of the new class. To construct the auxiliary matrix, only surviving individuals are assigned a number of recruits. This number of recruits is obtained from the fertility associated to the destination class of the individuals and is not directly given by the element in the fecundity matrix. The total number of recruits per group of group of reproductive individuals is then obtained and distributed among reproductives in the auxiliary matrix.

Using projection matrices assumes that all individuals within a class are identical and are all affected by the averaged vital rates in the matrix. However, the post-breeding projection matrix contains enough information to distinguish different types of individuals within the classes with an associated fecundity and this can be used to construct the auxiliary matrix.

For a post breeding census, fecundities and transitions are correlated, as the same survival probabilities and fertilities appear in different matrix elements. The fecundity matrix (***F***) is obtained from a transitions matrix (***T***) and a maternity matrix (***M***) (average number of recruits left per reproductive individual): ***F*** *=* ***M*** *x* ***T***. Individuals first survive and grow with probabilities given in ***T*** and then reproduce with maternities given in ***M***.

The fecundity and transitions matrices are given as input data and therefore, the maternity matrix may be obtained from ***M*** *=* ***F*** *x* ***T^+^***, where ***T^+^*** is the pseudoinverse of ***T***.

Inconsistences or incongruences may occur in projection matrices for post breeding censuses, particularly when the matrix is constructed collating information from different sources or when some matrix elements are guesses. When inconsistences exist, the above ***M*** matrix is an approximate solution: the best approach to the maternity values for those specific matrices of transitions and fecundity, but keeping transitions constant. The program uses this new maternity matrix. A new fecundity matrix is then obtained as ***F_new_*** *=* ***M*** *x* ***T***, and the new projection matrix is ***A*** *=* ***F_new_*** *+* ***T***. If inconsistences are small, ***F*** and ***F_new_*** are not very different and using ***M*** and ***F_new_*** does not introduce large distortions in calculations. If differences are large, a completely different transition matrix appears and results may be unreliable.

There may be good reasons to stick to the original matrix even though inconsistences are severe. The original matrix may be still used by following a different approach to estimate fertilities. The modification consists in that for classes with an associated fecundity, every reproductive individual reproduces with an average fertility, specific for the class, irrespective of the destination of the individual (staying or growing). The new average fertility is obtained by dividing the original fecundity of the class by the summation of the probabilities of ending in a reproductive class. In this way, the number of recruits produced by the class is identical to the expected by the original fecundity matrix, but every reproductive individual leaves the same number of recruits irrespective of their destination.

EXAMPLE

*Pre-breeding census*

Let’s assume the projection matrix obtained in a pre-breeding census after the study of 30, 30 and 20 individuals for classes 1 to 3 respectively: $\left( \begin{matrix} 0 & 1.8 & 3.6 \\ 0.4 & 0.2 & 0 \\ 0 & 0.3 & 0.6 \end{matrix} \right)$. Individuals in class 1 do not reproduce. Individuals at class 2 reproduce and leave an average of 1.8 recruits. That makes 54 recruits produced by the reproductives in class 2. Twenty percent of the recruits (10.8) are allocated to the 6 individuals staying in class 2, 30% of the recruits (16.2) are allocated to the 9 individuals promoting to class 3 and the remaining 50% of the recruits are allocated to the 15 individuals dying. Individuals at class 3 reproduce and leave an average of 3.6 recruits. From 72 recruits, 60% are allocated (43.2) to the 12 surviving individuals and 40% (28.8) to the 8 dead individuals. An example of how recruits are allocated in this latter case:

$\begin{matrix} Origin & 3 & 3 & 3 & 3 & 3 & 3 & 3 & 3 \\ Destination & 4 & 4 & 4 & 4 & 4 & 4 & 4 & 4 \\ Recruits & 4 & 4 & 4 & 4 & 3.8 & 3 & 3 & 3 \end{matrix}$

The final auxiliary matrix has this form:

$$\begin{matrix} 1 & & 1 & 2 & & 2 & 2 & 2 & & 2 & 2 & 2 & & 2 & & 3 & & 3 & 3 & & 3 & & 3 & 9 & \\ 2 & \ldots& 4 \ldots& 2 & \ldots& 2 & 2 & 3 & \ldots& 3 & 3 & 4 & \ldots& 4 & \ldots& 3 & \ldots& 3 & 3 & \ldots& 4 & \ldots& 4 & 4 & \ldots\\ 0 & \times12 & 0 \times18 & 2 & \times5 & 1.8 & 1 & 2 & \times7 & 1.2 & 1 & 2 & \times12 & 1 & \times3 & 4 & \times7 & 3.2 & 3 & \times4 & 4 & \times4 & 3.8 & 3 & \times3 \end{matrix}$$

*Post breeding census*

Let’s assume the same matrix and numbers of individuals as before. There are not inconsistences in the matrix and the fertility is 0, 0 and 6 for classes 1 to 3.

Individuals at class 1 do not reproduce and the columns for the auxiliary matrix are the same as in the pre-breeding case.

Individuals at class 2 dying or remaining do not reproduce. Nine individuals at class 2 and growing to class 3, reproduce with fertility associated to class 3 and produce a total of 54 recruits.

Individuals at class 3 and dying do not reproduce. Twelve individuals at class 3 and remaining reproduce with fertility associated to class 3 and produce 72 recruits.

The final auxiliary matrix has this form:

$$\begin{matrix} 1 & & 1 & & 2 & & 2 & & 2 & & 3 & & 3 & \\ 2 & \ldots& 4 & \ldots& 2 & \ldots& 3 & \ldots& 4 & \ldots& 3 & \ldots& 4 & \ldots\\ 0 & \times12 & 0 & \times18 & 0 & \times6 & 6 & \times9 & 0 & \times15 & 6 & \times12 & 0 & \times8 \end{matrix}$$

Inconsistences in the matrix entries

Let’s assume that the input matrix is $\left( \begin{matrix} 0 & 2 & 3.6 \\ 0.4 & 0.2 & 0 \\ 0 & 0.3 & 0.6 \end{matrix} \right)$

In a post-breeding census, that pattern of fecundities and transitions is not possible as the interpretation of the vital rates in the matrix is$\left( \begin{matrix} 0 & m_{3}S_{32} & m_{3}S_{33} \\ S_{21} & S_{22} & 0 \\ 0 & S_{32} & S_{33} \end{matrix} \right)$, where *m_3_* is a maternity and *S_ji_* are transition probabilities

An approximate solution for maternities is obtained from ***M*** *=* ***F*** *x* ***T^+^*** which generates a maternity matrix $\boldsymbol{M}=\left( \begin{matrix} 0 & 0.167 & 6.111 \\ 0 & 0 & 0 \\ 0 & 0 & 0 \end{matrix} \right)$ ,

a new fecundity matrix $\boldsymbol{F}_{\boldsymbol{new}}=\left( \begin{matrix} 0.067 & 1.867 & 3.667 \\ 0 & 0 & 0 \\ 0 & 0 & 0 \end{matrix} \right)$

and a new projection matrix $\boldsymbol{A}_{\boldsymbol{new}}=\left( \begin{matrix} 0.067 & 1.867 & 3.667 \\ 0.4 & 0.2 & 0 \\ 0 & 0.3 & 0.6 \end{matrix} \right)$.

If the new maternity scheme and projection matrices are acceptable, proceed as if this matrix was the original one.

If the adjusted maternity and fecundity matrices are not acceptable or the original matrix is considered the best approach, then a new maternity scheme is estimated from the original matrix. Fecundities for each class are divided by the summation of the transition probabilities leading to reproductive classes. For class 2 in the original matrix, because individuals staying do not reproduce, the only transition leading to a reproductive class is the probability to grow to class 3 and the new maternity is 2/0.3 = 6.667. The new maternity for class 3 remains the same (3.6/0.6 = 6).

With the new maternity scheme, individuals surviving during the projection interval and ending in a reproductive class reproduce with the new, adjusted maternity, irrespective of the destination class.
