# Supplementary material for: Pop‐Inference: An educational application to evaluate statistical differences among populations
Source: Ecol Evol. 2018 May 4;8(11):5224–30. doi: 10.1002/ece3.4010 (PMC6010711; doi:10.1002/ece3.4010)
Supplement: Supplementary file 3 [file ECE3-8-5224-s003.docx]

**APPENDIX S3. Example of a full output**

Data for the example come from unpublished population matrices of the intertidal barnacle *Chthamalus montagui* Southward from northern Spain. See details for sampling in Suárez & Arrontes (2008). The three matrices used as example are from 3 quadrats at different and distant sites. Census can be considered as post-breeding. Although barnacle populations are open populations, for the example they are treated as if they were closed populations. Newly recruited specimens are considered to come from reproductive individuals in the same quadrat.

The text only file with the data has this structure (for clarity data are truncated to the 3^rd^ decimal place):

0.024 0.152 0.639 1.214 0 0 0 0 262 0 [....Starts population 1]

0 0 0 0 0.148 0.440 0 0 125 1

0 0 0 0 0 0.112 0.655 0 122 1

0 0 0 0 0 0 0.114 0.793 58 1 [....Ends population 1]

0.052 0.292 0.697 1.170 0 0 0 0 154 0 [....Starts population 2]

0 0 0 0 0.376 0.483 0 0 118 1

0 0 0 0 0 0.381 0.752 0 121 1

0 0 0 0 0 0 0.198 0.915 59 1 [....Ends population 2]

0.033 0.242 0.675 1.114 0 0 0 0 203 0 [....Starts population 3]

0 0 0 0 0.241 0.519 0 0 127 1

0 0 0 0 0 0.291 0.638 0 105 1

0 0 0 0 0 0 0.238 0.878 66 1 [....Ends population 3]

The interpretation of the data is (for population 1):

| Fecundity matrix | | | | Transitions matrix | | | | N | R |
| --- | --- | --- | --- | --- | --- | --- | --- | --- | --- |
| 0.024 | 0.152 | 0.639 | 1.214 | 0 | 0 | 0 | 0 | 262 | 0 |
| 0 | 0 | 0 | 0 | 0.148 | 0.440 | 0 | 0 | 125 | 1 |
| 0 | 0 | 0 | 0 | 0 | 0.112 | 0.655 | 0 | 122 | 1 |
| 0 | 0 | 0 | 0 | 0 | 0 | 0.114 | 0.793 | 58 | 1 |

Where N is the number of individuals at stages 1 to 4 (top to bottom) and R states whether stages 1 to 4 are reproductive (1) or not (0).

Most of the sections are self explained. For confidence intervals the first value of the pair is the low limit and the second value is the high limit. For the reproductive value and the stable stage structure, there are four columns. The 2 first columns are the low (left) and high (right) limits for the 95% confidence intervals for the vital rate of each of the four stages in the population. The two other columns are for the 90% confidence intervals.

For planned comparisons, population 1 was compared with populations 2 and 3 pooled

For power analysis, the output have the same structure but confidence intervals and differences are shown for all evaluated densities.

Cited paper:

Suárez, R. & Arrontes, J. (2008). Population dynamics of the barnacle *Chthamalus montagui* at two spatial and temporal scales in northern Spain. *Marine Biology*, 155, 363-374.

File with input data: Matrix3.txt

Results file: E:\Data\Demography Data\output1.txt

Sampling scheme:

The number of individuals in each class was obtained at random

Number of simulations: 10000

-----------------------------

Population no. 1:

0.0248 0.1524 0.6392 1.2149

0.1489 0.4400 0.0000 0.0000

0.0000 0.1120 0.6557 0.0000

0.0000 0.0000 0.1148 0.7931

Fecundity matrix

0.0248 0.1524 0.6392 1.2149

0.0000 0.0000 0.0000 0.0000

0.0000 0.0000 0.0000 0.0000

0.0000 0.0000 0.0000 0.0000

Transitions matrix

0.0000 0.0000 0.0000 0.0000

0.1489 0.4400 0.0000 0.0000

0.0000 0.1120 0.6557 0.0000

0.0000 0.0000 0.1148 0.7931

HISTORIES:

Origin 1 1 1 2 2 2 2 2 3 3 3 3 3 4 4 4

Destination 2 2 5 2 2 3 3 5 3 3 4 4 5 4 4 5

No. Recruits 0 1 0 0 1 0 1 0 0 1 1 2 0 1 2 0

...in stages 0 1 0 0 1 0 1 0 0 1 1 1 0 1 1 0

Individuals 32 7 223 45 10 4 10 56 23 57 6 8 28 21 25 12

ORIGINAL LAMBDA: 0.8428

95% Confidence interval (CI): 0.7730 0.9227

90% Confidence interval (CI): 0.7841 0.9107

Simulated median Lambda: 0.8458

Intervals with corrected bias:

Corrected 95% CI: 0.7666 0.9175

Corrected 90% CI: 0.7782 0.9049

NET REPRODUCTIVE RATE (Ro): 0.1789

Simulated median Ro: 0.1779

95% CI: 0.0973 0.3182

90% CI: 0.1086 0.2883

GENERATION TIME (GT): 10.0654

Simulated median GT: 10.2736

95% CI: 7.5575 17.2467

90% CI: 7.8654 15.5842

OBSERVED STAGE STRUCTURE:

0.4621

0.2205

0.2152

0.1023

Distance to SSS: 0.1545

Probability (p-value): 0.0001

STABLE STAGE STRUCTURE (SSS):

0.4759

0.1759

0.1053

0.2430

Median SSS:

0.4754

0.1735

0.1039

0.2427

95% and 90% CIs:

0.4284 0.5164 0.4366 0.5100

0.1258 0.2352 0.1327 0.2239

0.0489 0.2002 0.0556 0.1809

0.1720 0.2984 0.1841 0.2905

REPRODUCTIVE VALUE (RV):

0.0203

0.1114

0.3731

0.4953

Median RV:

0.0195

0.1092

0.3707

0.4967

95% and 90% CIs:

0.0090 0.0354 0.0103 0.0325

0.0547 0.1807 0.0621 0.1676

0.2545 0.5066 0.2738 0.4853

0.3081 0.6664 0.3381 0.6375

ELASTICITY MATRIX:

0.0015 0.0034 0.0086 0.0376

0.0496 0.0542 0.0000 0.0000

0.0000 0.0462 0.1618 0.0000

0.0000 0.0000 0.0376 0.5996

SENSITIVITY MATRIX:

0.0511 0.0189 0.0113 0.0261

0.2807 0.1037 0.0621 0.1433

0.9401 0.3474 0.2080 0.4800

1.2481 0.4612 0.2761 0.6372

-----------------------------

Population no. 2:

0.0524 0.2922 0.6975 1.1705

0.3766 0.4831 0.0000 0.0000

0.0000 0.3814 0.7521 0.0000

0.0000 0.0000 0.1983 0.9153

Fecundity matrix

0.0524 0.2922 0.6975 1.1705

0.0000 0.0000 0.0000 0.0000

0.0000 0.0000 0.0000 0.0000

0.0000 0.0000 0.0000 0.0000

Transitions matrix

0.0000 0.0000 0.0000 0.0000

0.3766 0.4831 0.0000 0.0000

0.0000 0.3814 0.7521 0.0000

0.0000 0.0000 0.1983 0.9153

HISTORIES:

Origin 1 1 1 2 2 2 2 2 3 3 3 3 3 4 4 4

Destination 2 2 5 2 2 3 3 5 3 3 4 4 5 4 4 5

No. Recruits 0 1 0 0 1 0 1 0 0 1 1 2 0 1 2 0

...in stages 0 1 0 0 1 0 1 0 0 1 1 1 0 1 1 0

Individuals 49 9 96 49 8 18 27 16 37 54 17 7 6 38 16 5

ORIGINAL LAMBDA: 1.1473

95% Confidence interval (CI): 1.0933 1.1984

90% Confidence interval (CI): 1.1013 1.1902

Simulated median Lambda: 1.1473

Intervals with corrected bias:

Corrected 95% CI: 1.0933 1.1983

Corrected 90% CI: 1.1010 1.1901

NET REPRODUCTIVE RATE (Ro): 4.1169

Simulated median Ro: 4.2192

95% CI: 2.2555 13.9830

90% CI: 2.4660 10.1137

GENERATION TIME (GT): 10.3010

Simulated median GT: 10.4904

95% CI: 8.0603 16.4989

90% CI: 8.3584 14.6975

OBSERVED STAGE STRUCTURE:

0.3407

0.2611

0.2677

0.1305

Distance to SSS: 0.0972

Probability (p-value): 0.0001

STABLE STAGE STRUCTURE (SSS):

0.3873

0.2196

0.2119

0.1812

Median SSS:

0.3868

0.2195

0.2115

0.1805

95% and 90% CIs:

0.3611 0.4127 0.3650 0.4079

0.1831 0.2590 0.1889 0.2524

0.1697 0.2647 0.1755 0.2553

0.1420 0.2178 0.1480 0.2117

REPRODUCTIVE VALUE (RV):

0.0755

0.2194

0.3243

0.3808

Median RV:

0.0750

0.2189

0.3243

0.3816

95% and 90% CIs:

0.0603 0.0922 0.0624 0.0892

0.1884 0.2531 0.1928 0.2472

0.2939 0.3544 0.2990 0.3496

0.3212 0.4383 0.3313 0.4281

ELASTICITY MATRIX:

0.0062 0.0196 0.0452 0.0649

0.1297 0.0943 0.0000 0.0000

0.0000 0.1100 0.2094 0.0000

0.0000 0.0000 0.0649 0.2558

SENSITIVITY MATRIX:

0.1359 0.0770 0.0743 0.0636

0.3950 0.2240 0.2161 0.1848

0.5839 0.3311 0.3195 0.2731

0.6855 0.3887 0.3751 0.3207

-----------------------------

Population no. 3:

0.0333 0.2422 0.6754 1.1147

0.2414 0.5197 0.0000 0.0000

0.0000 0.2913 0.6381 0.0000

0.0000 0.0000 0.2381 0.8788

Fecundity matrix

0.0333 0.2422 0.6754 1.1147

0.0000 0.0000 0.0000 0.0000

0.0000 0.0000 0.0000 0.0000

0.0000 0.0000 0.0000 0.0000

Transitions matrix

0.0000 0.0000 0.0000 0.0000

0.2414 0.5197 0.0000 0.0000

0.0000 0.2913 0.6381 0.0000

0.0000 0.0000 0.2381 0.8788

HISTORIES:

Origin 1 1 1 2 2 2 2 2 3 3 3 3 3 4 4 4

Destination 2 2 5 2 2 3 3 5 3 3 4 4 5 4 4 5

No. Recruits 0 1 0 0 1 0 1 0 0 1 1 2 0 1 2 0

...in stages 0 1 0 0 1 0 1 0 0 1 1 1 0 1 1 0

Individuals 42 7 154 56 10 15 22 24 27 40 18 7 13 42 16 8

ORIGINAL LAMBDA: 1.0271

95% Confidence interval (CI): 0.9668 1.0823

90% Confidence interval (CI): 0.9779 1.0743

Simulated median Lambda: 1.0278

Intervals with corrected bias:

Corrected 95% CI: 0.9648 1.0809

Corrected 90% CI: 0.9759 1.0730

NET REPRODUCTIVE RATE (Ro): 1.3140

Simulated median Ro: 1.3288

95% CI: 0.7408 3.0255

90% CI: 0.8173 2.5248

GENERATION TIME (GT): 10.2307

Simulated median GT: 10.3855

95% CI: 7.8959 16.1833

90% CI: 8.2027 14.7976

OBSERVED STAGE STRUCTURE:

0.4052

0.2535

0.2096

0.1317

Distance to SSS: 0.1170

Probability (p-value): 0.0001

STABLE STAGE STRUCTURE (SSS):

0.4159

0.1979

0.1482

0.2380

Median SSS:

0.4151

0.1974

0.1471

0.2378

95% and 90% CIs:

0.3851 0.4446 0.3900 0.4401

0.1574 0.2453 0.1635 0.2372

0.1075 0.1992 0.1132 0.1894

0.1937 0.2800 0.2016 0.2731

REPRODUCTIVE VALUE (RV):

0.0527

0.2170

0.3341

0.3962

Median RV:

0.0521

0.2160

0.3339

0.3970

95% and 90% CIs:

0.0381 0.0684 0.0402 0.0658

0.1718 0.2620 0.1789 0.2554

0.2931 0.3732 0.3011 0.3666

0.3232 0.4737 0.3348 0.4607

ELASTICITY MATRIX:

0.0034 0.0118 0.0246 0.0652

0.1016 0.1041 0.0000 0.0000

0.0000 0.0899 0.1474 0.0000

0.0000 0.0000 0.0652 0.3867

SENSITIVITY MATRIX:

0.1050 0.0500 0.0374 0.0601

0.4325 0.2058 0.1541 0.2475

0.6658 0.3168 0.2373 0.3810

0.7897 0.3757 0.2814 0.4519

-----------------------------

AMONG ALL POPULATIONS. GLOBAL CONTRASTS:

Asymptotic growth rate (Lambda)

Observed Sum of Squares: 0.0470

Probability (p-value): 0.0001

Net reproductive rate (Ro)

Observed Sum of Squares: 8.2178

Probability (p-value): 0.0016

Generation Time (GT)

Observed Sum of Squares: 0.0293

Probability (p-value): 0.9948

Stable Stage Structure (SSS)

Average distance: 0.0839

Probability (p-value): 0.0245

Reproductive value (RV)

Average distance: 0.1004

Probability (p-value): 0.0311

Histories

Average distance: 0.1746

Probability (p-value): 0.0001

DIFFERENCES BETWEEN POPULATION 1 AND POPULATION 2:

Difference between lambdas: 0.3044

Probability (p-value): 0.0001

Difference between Ro´s: 3.9381

Probability (p-value): 0.0008

Difference between GTs: 0.2356

Probability (p-value): 0.9206

Distance between SSSs: 0.1504

Probability (p-value): 0.0037

Distance between RVs: 0.1632

Probability (p-value): 0.0151

Distance between Histories: 0.2992

Probability (p-value): 0.0001

DIFFERENCES BETWEEN POPULATION 1 AND POPULATION 3:

Difference between lambdas: 0.1842

Probability (p-value): 0.0001

Difference between Ro´s: 1.1351

Probability (p-value): 0.0296

Difference between GTs: 0.1653

Probability (p-value): 0.9460

Distance between SSSs: 0.0649

Probability (p-value): 0.2510

Distance between RVs: 0.1380

Probability (p-value): 0.0328

Distance between Histories: 0.1984

Probability (p-value): 0.0006

DIFFERENCES BETWEEN POPULATION 2 AND POPULATION 3:

Difference between lambdas: 0.1202

Probability (p-value): 0.0067

Difference between Ro´s: 2.8029

Probability (p-value): 0.0023

Difference between GTs: 0.0703

Probability (p-value): 0.9781

Distance between SSSs: 0.0854

Probability (p-value): 0.1348

Distance between RVs: 0.0252

Probability (p-value): 0.8661

Distance between Histories: 0.1307

Probability (p-value): 0.0899

PLANNED COMPARISONS:

COMPARISON 1:

Group of populations 'A', formed by populations: 1

Group of populations 'B', formed by populations: 2 3

PAIRWISE COMPARISONS

(Probability of the observed distance or difference)

Among lambdas (A and B): 0.0001

Among Ro´s (A and B): 0.0010

Among GT´s (A and B): 0.9511

Among SSSs (A and B): 0.0163

Among RVs (A and B): 0.0051

Among histories (A and B): 0.1123

LIFE TABLE RESPONSE EXPERIMENT (LTRE):

(Fixed factors)

Population no. 1:

-0.0010 -0.0027 -0.0008 0.0020

-0.0380 -0.0064 0.0000 0.0000

0.0000 -0.0523 -0.0067 0.0000

0.0000 0.0000 -0.0223 -0.0351

Population no. 2:

0.0019 0.0041 0.0015 0.0002

0.0479 0.0005 0.0000 0.0000

0.0000 0.0403 0.0211 0.0000

0.0000 0.0000 0.0053 0.0194

Population no. 3:

-0.0004 0.0007 0.0002 -0.0029

-0.0059 0.0077 0.0000 0.0000

0.0000 0.0098 -0.0112 0.0000

0.0000 0.0000 0.0166 0.0073

Identification of vital rates:

1 5 9 13

2 6 10 14

3 7 11 15

4 8 12 16

**OUTPUT FOR POWER ANALYSIS**

Name of file with the data: Matrix3.txt

Results file: E:\Datos\Demography Data\Results\paper2.txt

Number of individuals used in each density (0 = original data)

Density Pop 1 Pop 2 Pop 3

0 567 452 501

1 680 543 601

2 817 651 721

3 980 781 865

4 1175 937 1039

5 1411 1125 1246

Confidence intervals for lambda:

Population number 1:

No. Low 95% Up 95% Low 90% Up 90%

567 0.77391 0.92231 0.78414 0.90933

680 0.78604 0.92183 0.79540 0.91036

817 0.78369 0.90911 0.79423 0.89969

980 0.78462 0.90128 0.79439 0.89128

1175 0.79145 0.89791 0.79958 0.88912

1411 0.79615 0.89303 0.80325 0.88376

Population number 2:

No. Low 95% Up 95% Low 90% Up 90%

452 1.09310 1.19638 1.10214 1.18933

543 1.09966 1.19429 1.10860 1.18708

651 1.10654 1.19264 1.11380 1.18586

781 1.10512 1.18523 1.11159 1.17931

937 1.11219 1.18411 1.11785 1.17855

1125 1.11471 1.18070 1.12015 1.17566

Population number 3:

No. Low 95% Up 95% Low 90% Up 90%

501 0.97004 1.08315 0.97854 1.07484

601 0.96988 1.07577 0.97945 1.06709

721 0.97458 1.06939 0.98215 1.06270

865 0.98257 1.06872 0.98947 1.06194

1039 0.98707 1.06589 0.99331 1.05970

1246 0.99081 1.06223 0.99619 1.05655

Confidence intervals for the Net Reproductive Rate:

Population number 1:

No. Low 95% Up 95% Low 90% Up 90%

567 0.09902 0.31725 0.10947 0.28537

680 0.10863 0.31857 0.11850 0.28935

817 0.10913 0.28405 0.11780 0.26175

980 0.11167 0.27108 0.12114 0.25052

1175 0.12027 0.26362 0.12806 0.24739

1411 0.12355 0.25446 0.13141 0.23877

Population number 2:

No. Low 95% Up 95% Low 90% Up 90%

452 2.24787 13.59489 2.46022 9.87551

543 2.36934 11.52418 2.58419 9.48080

651 2.54769 10.99374 2.76019 8.97216

781 2.48040 8.67078 2.66987 7.39885

937 2.71701 9.00108 2.90219 7.67471

1125 2.80508 8.11440 2.96918 7.09215

Population number 3:

No. Low 95% Up 95% Low 90% Up 90%

501 0.75925 3.03364 0.82208 2.58611

601 0.76381 2.60804 0.82561 2.22967

721 0.79162 2.34785 0.84724 2.08329

865 0.84862 2.29359 0.90646 2.05903

1039 0.88624 2.15798 0.93891 1.96675

1246 0.91830 2.05475 0.96496 1.89526

Confidence intervals for the Generation Time:

Population number 1:

No. Low 95% Up 95% Low 90% Up 90%

567 7.54772 17.35382 7.87849 15.63759

680 7.84465 16.77771 8.14770 15.28660

817 7.89806 15.81194 8.17389 14.62250

980 7.91954 14.68158 8.17283 13.61492

1175 8.03755 14.05189 8.29555 13.23947

1411 8.19231 13.56147 8.42369 12.78640

Population number 2:

No. Low 95% Up 95% Low 90% Up 90%

452 8.05033 16.36164 8.37127 14.72592

543 8.21888 15.40930 8.49181 14.29813

651 8.39182 15.00468 8.65987 13.96621

781 8.38122 13.92804 8.63260 13.12896

937 8.69852 14.02237 8.93100 13.29682

1125 8.81308 13.59564 9.04396 12.92394

Population number 3:

No. Low 95% Up 95% Low 90% Up 90%

501 7.92700 16.24460 8.20971 14.66790

601 7.93509 14.75869 8.21533 13.75501

721 8.12045 14.18843 8.35484 13.24691

865 8.29591 13.83033 8.54546 13.05903

1039 8.38433 13.22245 8.62640 12.68175

1246 8.58236 13.11187 8.79998 12.52444

Confidence intervals for SSS:

Population number 1:

No. Lo 95% Up 95% Lo 90% Up 90%

567 0.4300 0.5162 0.4369 0.5102

0.1274 0.2351 0.1339 0.2242

0.0483 0.2010 0.0542 0.1821

0.1697 0.2994 0.1830 0.2908

680 0.4347 0.5133 0.4413 0.5078

0.1296 0.2279 0.1358 0.2173

0.0515 0.1874 0.0583 0.1702

0.1806 0.2945 0.1915 0.2874

817 0.4381 0.5112 0.4445 0.5059

0.1330 0.2224 0.1392 0.2139

0.0533 0.1794 0.0594 0.1635

0.1860 0.2917 0.1960 0.2853

980 0.4403 0.5072 0.4461 0.5023

0.1377 0.2187 0.1430 0.2115

0.0592 0.1743 0.0651 0.1612

0.1896 0.2869 0.1986 0.2804

1175 0.4422 0.5044 0.4480 0.4997

0.1409 0.2174 0.1460 0.2095

0.0628 0.1677 0.0685 0.1573

0.1923 0.2827 0.2013 0.2758

1411 0.4465 0.5013 0.4513 0.4974

0.1440 0.2121 0.1486 0.2053

0.0664 0.1622 0.0719 0.1510

0.1983 0.2788 0.2065 0.2735

Population number 2:

No. Lo 95% Up 95% Lo 90% Up 90%

452 0.3607 0.4120 0.3650 0.4075

0.1833 0.2598 0.1890 0.2529

0.1694 0.2665 0.1756 0.2563

0.1408 0.2177 0.1478 0.2120

543 0.3629 0.4100 0.3668 0.4063

0.1866 0.2558 0.1918 0.2504

0.1722 0.2593 0.1775 0.2505

0.1453 0.2156 0.1506 0.2096

651 0.3666 0.4084 0.3698 0.4049

0.1888 0.2518 0.1937 0.2466

0.1740 0.2546 0.1797 0.2473

0.1495 0.2129 0.1546 0.2078

781 0.3663 0.4054 0.3695 0.4025

0.1917 0.2506 0.1965 0.2452

0.1798 0.2530 0.1846 0.2467

0.1499 0.2080 0.1551 0.2037

937 0.3704 0.4055 0.3728 0.4027

0.1936 0.2463 0.1972 0.2423

0.1801 0.2456 0.1846 0.2399

0.1553 0.2080 0.1594 0.2040

1125 0.3715 0.4039 0.3741 0.4014

0.1959 0.2442 0.1990 0.2400

0.1822 0.2431 0.1864 0.2366

0.1575 0.2060 0.1615 0.2024

Population number 3:

No. Lo 95% Up 95% Lo 90% Up 90%

501 0.3852 0.4451 0.3896 0.4404

0.1572 0.2445 0.1634 0.2363

0.1081 0.2011 0.1139 0.1902

0.1928 0.2796 0.2012 0.2726

601 0.3872 0.4406 0.3913 0.4367

0.1619 0.2422 0.1676 0.2349

0.1113 0.1950 0.1168 0.1867

0.1973 0.2749 0.2035 0.2690

721 0.3904 0.4394 0.3944 0.4357

0.1630 0.2361 0.1690 0.2299

0.1144 0.1897 0.1191 0.1827

0.2007 0.2723 0.2071 0.2668

865 0.3920 0.4373 0.3959 0.4335

0.1671 0.2339 0.1719 0.2280

0.1171 0.1865 0.1218 0.1800

0.2041 0.2690 0.2094 0.2639

1039 0.3940 0.4357 0.3973 0.4324

0.1698 0.2309 0.1741 0.2251

0.1206 0.1847 0.1247 0.1781

0.2060 0.2652 0.2115 0.2611

1246 0.3969 0.4338 0.4003 0.4308

0.1715 0.2264 0.1754 0.2217

0.1220 0.1791 0.1260 0.1737

0.2102 0.2643 0.2152 0.2600

Confidence intervals for RV:

Population number 1:

No. Lo 95% Up 95% Lo 90% Up 90%

567 0.0087 0.0353 0.0101 0.0328

0.0534 0.1824 0.0604 0.1688

0.2572 0.5103 0.2750 0.4841

0.3077 0.6638 0.3400 0.6392

680 0.0096 0.0338 0.0109 0.0311

0.0584 0.1734 0.0660 0.1617

0.2666 0.4917 0.2831 0.4687

0.3318 0.6510 0.3580 0.6255

817 0.0099 0.0321 0.0111 0.0300

0.0598 0.1671 0.0665 0.1562

0.2691 0.4773 0.2841 0.4576

0.3474 0.6443 0.3756 0.6254

980 0.0110 0.0318 0.0123 0.0299

0.0658 0.1660 0.0726 0.1565

0.2834 0.4732 0.2971 0.4546

0.3541 0.6279 0.3775 0.6056

1175 0.0119 0.0310 0.0130 0.0292

0.0710 0.1617 0.0763 0.1526

0.2924 0.4659 0.3055 0.4498

0.3621 0.6116 0.3836 0.5941

1411 0.0126 0.0300 0.0136 0.0284

0.0738 0.1562 0.0793 0.1485

0.2989 0.4558 0.3108 0.4421

0.3758 0.6031 0.3951 0.5857

Population number 2:

No. Lo 95% Up 95% Lo 90% Up 90%

452 0.0603 0.0918 0.0626 0.0889

0.1878 0.2534 0.1927 0.2480

0.2943 0.3554 0.2995 0.3500

0.3201 0.4374 0.3311 0.4282

543 0.0613 0.0904 0.0636 0.0876

0.1905 0.2497 0.1951 0.2445

0.2969 0.3520 0.3015 0.3475

0.3271 0.4327 0.3354 0.4238

651 0.0628 0.0890 0.0646 0.0866

0.1925 0.2468 0.1965 0.2424

0.3006 0.3502 0.3044 0.3467

0.3310 0.4276 0.3397 0.4199

781 0.0640 0.0883 0.0658 0.0862

0.1957 0.2462 0.1998 0.2416

0.2994 0.3461 0.3033 0.3420

0.3349 0.4258 0.3418 0.4185

937 0.0641 0.0859 0.0658 0.0841

0.1953 0.2407 0.1986 0.2368

0.3038 0.3462 0.3072 0.3429

0.3419 0.4218 0.3492 0.4157

1125 0.0650 0.0849 0.0666 0.0833

0.1980 0.2397 0.2013 0.2361

0.3052 0.3428 0.3078 0.3396

0.3461 0.4198 0.3524 0.4135

Population number 3:

No. Lo 95% Up 95% Lo 90% Up 90%

501 0.0380 0.0686 0.0402 0.0660

0.1738 0.2620 0.1804 0.2544

0.2941 0.3729 0.3006 0.3657

0.3225 0.4721 0.3350 0.4600

601 0.0399 0.0679 0.0417 0.0653

0.1770 0.2607 0.1832 0.2534

0.2972 0.3693 0.3032 0.3637

0.3261 0.4666 0.3372 0.4552

721 0.0406 0.0661 0.0423 0.0637

0.1817 0.2552 0.1868 0.2497

0.3018 0.3669 0.3069 0.3613

0.3329 0.4571 0.3423 0.4463

865 0.0418 0.0649 0.0435 0.0631

0.1843 0.2520 0.1893 0.2461

0.3029 0.3616 0.3077 0.3572

0.3407 0.4539 0.3500 0.4451

1039 0.0430 0.0644 0.0446 0.0625

0.1886 0.2513 0.1932 0.2455

0.3061 0.3603 0.3110 0.3559

0.3426 0.4464 0.3512 0.4380

1246 0.0435 0.0626 0.0448 0.0609

0.1891 0.2449 0.1932 0.2407

0.3094 0.3593 0.3139 0.3553

0.3485 0.4436 0.3568 0.4353

-----------------------------

DIFFERENCES BETWEEN POPULATION 1 AND POPULATION 2:

Differences between lambdas:

No.1 No.2 P-value Power

567 452 0.0001 1.0000

680 543 0.0001 1.0000

817 651 0.0001 1.0000

980 781 0.0001 1.0000

1175 937 0.0001 1.0000

1411 1125 0.0001 1.0000

Differences between net reproductive rates:

No.1 No.2 P-value Power

567 452 0.0008 1.0000

680 543 0.0002 1.0000

817 651 0.0001 1.0000

980 781 0.0001 1.0000

1175 937 0.0001 1.0000

1411 1125 0.0001 1.0000

Differences between generation times:

No.1 No.2 P-value Power

567 452 0.9204 0.0796

680 543 0.9168 0.0908

817 651 0.9055 0.0878

980 781 0.9007 0.0770

1175 937 0.8875 0.0916

1411 1125 0.8791 0.0879

Distance in SSS:

No.1 No.2 P-value Power

567 452 0.0048 0.7823

680 543 0.0015 0.8617

817 651 0.0005 0.9089

980 781 0.0003 0.9372

1175 937 0.0002 0.9627

1411 1125 0.0001 0.9848

Distance in RV:

No.1 No.2 P-value Power

567 452 0.0143 0.8080

680 543 0.0080 0.8984

817 651 0.0042 0.9548

980 781 0.0019 0.9803

1175 937 0.0002 0.9919

1411 1125 0.0002 0.9979

Distance in Histories:

No.1 No.2 P-value Power

567 452 0.0001 0.9932

680 543 0.0001 0.9955

817 651 0.0001 0.9983

980 781 0.0001 0.9991

1175 937 0.0001 0.9991

1411 1125 0.0001 0.9995

DIFFERENCES BETWEEN POPULATION 1 AND POPULATION 3:

Differences between lambdas:

No.1 No.3 P-value Power

567 501 0.0001 0.9792

680 601 0.0001 0.9866

817 721 0.0001 0.9964

980 865 0.0001 0.9999

1175 1039 0.0001 0.9999

1411 1246 0.0001 1.0000

Differences between net reproductive rates:

No.1 No.3 P-value Power

567 501 0.0324 0.6835

680 601 0.0179 0.7709

817 721 0.0102 0.8946

980 865 0.0033 0.9789

1175 1039 0.0025 0.9953

1411 1246 0.0005 0.9993

Differences between generation times:

No.1 No.3 P-value Power

567 501 0.9449 0.0861

680 601 0.9380 0.0971

817 721 0.9349 0.0880

980 865 0.9279 0.0837

1175 1039 0.9170 0.0868

1411 1246 0.9125 0.0802

Distance in SSS:

No.1 No.3 P-value Power

567 501 0.2576 0.2891

680 601 0.1927 0.3733

817 721 0.1536 0.4259

980 865 0.1156 0.4286

1175 1039 0.0722 0.5112

1411 1246 0.0472 0.5552

Distance in RV:

No.1 No.3 P-value Power

567 501 0.0378 0.6697

680 601 0.0191 0.7859

817 721 0.0111 0.8557

980 865 0.0066 0.9057

1175 1039 0.0020 0.9580

1411 1246 0.0010 0.9770

Distance in Histories:

No.1 No.3 P-value Power

567 501 0.0003 0.6022

680 601 0.0003 0.5849

817 721 0.0001 0.6848

980 865 0.0001 0.7365

1175 1039 0.0001 0.8148

1411 1246 0.0001 0.8794

DIFFERENCES BETWEEN POPULATION 2 AND POPULATION 3:

Differences between lambdas:

No.2 No.3 P-value Power

452 501 0.0070 0.8023

543 601 0.0020 0.9005

651 721 0.0009 0.9539

781 865 0.0004 0.9617

937 1039 0.0001 0.9853

1125 1246 0.0001 0.9961

Differences between net reproductive rates:

No.2 No.3 P-value Power

452 501 0.0025 0.9126

543 601 0.0007 0.9619

651 721 0.0003 0.9853

781 865 0.0002 0.9869

937 1039 0.0001 0.9958

1125 1246 0.0001 0.9983

Differences between generation times:

No.2 No.3 P-value Power

452 501 0.9793 0.0567

543 601 0.9734 0.0530

651 721 0.9760 0.0499

781 865 0.9707 0.0492

937 1039 0.9651 0.0571

1125 1246 0.9634 0.0560

Distance in SSS:

No.2 No.3 P-value Power

452 501 0.1414 0.3175

543 601 0.0943 0.3788

651 721 0.0629 0.4354

781 865 0.0435 0.5628

937 1039 0.0262 0.6087

1125 1246 0.0143 0.7195

Distance in RV:

No.2 No.3 P-value Power

452 501 0.8557 0.0104

543 601 0.8358 0.0085

651 721 0.8000 0.0091

781 865 0.7650 0.0093

937 1039 0.7228 0.0096

1125 1246 0.6738 0.0117

Distance in Histories:

No.2 No.3 P-value Power

452 501 0.0902 0.5495

543 601 0.0388 0.6132

651 721 0.0106 0.6309

781 865 0.0067 0.6513

937 1039 0.0016 0.6692

1125 1246 0.0006 0.6938

------------------------------------------------

DIFFERENCES AMONG ALL POPULATIONS. GLOBAL CONTRASTS

Lambda:

Density P-value Power

0 0.0001 1.0000

1 0.0001 1.0000

2 0.0001 1.0000

3 0.0001 1.0000

4 0.0001 1.0000

5 0.0001 1.0000

Net reproductive rate, Ro:

Density P-value Power

0 0.0016 0.9999

1 0.0003 0.9999

2 0.0003 1.0000

3 0.0001 1.0000

4 0.0001 1.0000

5 0.0001 1.0000

Generation time:

Density P-value Power

0 0.9943 0.0811

1 0.9932 0.0906

2 0.9930 0.0847

3 0.9915 0.0756

4 0.9876 0.0902

5 0.9894 0.0863

Stable stage structure:

Density P-value Power

0 0.0274 0.7372

1 0.0104 0.8450

2 0.0035 0.9042

3 0.0014 0.9401

4 0.0002 0.9697

5 0.0001 0.9894

Reproductive value:

Density P-value Power

0 0.0297 0.7361

1 0.0139 0.8483

2 0.0066 0.9171

3 0.0028 0.9566

4 0.0006 0.9830

5 0.0003 0.9945

Histories:

Density P-value Power

0 0.0001 0.9799

1 0.0001 0.9859

2 0.0001 0.9930

3 0.0001 0.9976

4 0.0001 0.9974

5 0.0001 0.9991
